# Geography shapes the genomics and antimicrobial resistance of *Salmonella enterica* Serovar Enteritidis isolated from humans

**DOI:** 10.1038/s41598-022-24150-4

**Published:** 2023-01-24

**Authors:** Guojie Cao, Shaohua Zhao, Dai Kuang, Chih-Hao Hsu, Lanlan Yin, Yan Luo, Zhao Chen, Xuebin Xu, Errol Strain, Patrick McDermott, Marc Allard, Eric Brown, Jianghong Meng, Jie Zheng

**Affiliations:** 1 Center for Food Safety and Applied Nutrition, U.S. Food and Drug Administration, College Park, MD 20740 USA; 2 Center for Veterinary Medicine, U.S. Food and Drug Administration, Laurel, MD 20708 USA; 3 Ruijin Hospital, School of Medicine, Shanghai Jiao Tong University, Shanghai, China; 4 Joint Institute for Food Safety and Applied Nutrition, Center for Food Safety & Security Systems, Department of Nutrition and Food Science, University of Maryland, College Park, MD USA; 5 Shanghai Municipal Center for Disease Control and Prevention, Shanghai, China

**Keywords:** Bacterial genomics, Antimicrobial resistance

## Abstract

Multidrug-resistant (MDR) *Salmonella* has been a long-standing challenge in public health and food safety. The prevalence of MDR *S*. Enteritidis, especially isolated from humans, in China is significantly higher than those from the U.S. and other countries. A dataset of 197 *S*. Enteritidis genomes, including 16 sequenced clinical isolates from China and 181 downloaded genomes of human isolates from the U.S., Europe, and Africa, was analyzed for genomic diversity, virulence potential, and antimicrobial resistance (AMR). Phylogenomic analyses identified four major well-supported clades (I–IV). While AMR genotype in the majority of isolates in clades I and IV displayed as pan-susceptible, 81.8% (9/11) and 22.4% (13/58) of isolates in clades III and II were MDR, respectively. It is noted that 77% (10/13) of MDR isolates in clade II were from China. The most common antimicrobial resistance genes (ARGs) carried by the Chinese isolates were *aph(3′)-IIa*, *bla*_CTX-M-55_, and *bla*_TEM-1B_, whereas *bla*_TEM-1B_, *sul1*, *sul2*, *drfA7*, *aph(3")-Ib*/*strA*, and *aph(6)-Id*/str*B* were most often identified in those from Africa (clade III). Among the 14 plasmid types identified, IncX1 and IncFII(pHN7A8) were found exclusively in the Chinese MDR isolates, while IncQ1 was highly associated with the African MDR isolates. The *spvRABCD* virulence operon was present in 94.9% (187/197) of isolates tested and was highly associated with both the IncF (IncFII and IncFIB) plasmids. In addition, phylogenetic differences in distribution of *Salmonella* pathogenicity islands (SPIs), prophages and other accessory genes were also noted. Taken together, these findings provide new insights into the molecular mechanisms underpinning diversification of MDR *S*. Enteritidis.

## Introduction

*Salmonella* is a leading bacterial cause of diarrheal disease in humans, leading to an estimated 93.8 million gastroenteritis cases and 155,000 deaths annually worldwide^1^. *Salmonella* Enteritidis (*S*. Enteritidis), a host-promiscuous serotype along with *S*. Typhimurium, are consistently ranked as the top two serotypes most frequently associated with human disease around the world^2^. The two serovars account for > 40% of human salmonellosis worldwide -> 30% in China^3^, and approximately 20% in the U.S.^4^. The clinical outcome of *S*. Enteritidis infection varies, ranging from self-limiting enterocolitis and acute pancreatitis to invasive infections with unusually high fatality^5^.

The continued emergence and expansion of antimicrobial resistance (AMR) in *Salmonella* has constitutes a serious public health challenge^4^. AMR in *Salmonella* varies not only by serotype, but also by source and geographical location. For example, AMR in *S*. Enteritidis is relatively low compared to many other important serotypes, such as *S*. Typhimurium, *S*. Newport, and I.4.[5].0.12:i:-. However, the dynamic of resistance in *S*. Enteritidis is changing over time in the U.S. Recently, the CDC reported a significant surge in resistance to clinically important antimicrobials (ampicillin and ceftriaxone or nonsusceptibility to ciprofloxacin) occurred among *S*. Enteritidis in 2015–2016 as compared to 2004–2008^4^. The prevalence of multiple drug resistance (MDR), (i.e. resistance to at least two classes of antimicrobials) in *S*. Enteritidis varied greatly from different regions and countries as well: 2.2% in the U.S.^4^, 3.2% in Europe^6^, 42% in sub-Saharan Africa^5^, and 40–81% in China^7,8^.

Plasmid serves as a major mobile genetic element in the spread of AMR^10^. A variety of plasmids have been reported to carry AMR genes and confer MDR in a variety of *Salmonella* serotypes. The most prevalent plasmid replicons include IncF, IncI, IncA/C, IncX, IncH, and Col^11,12^. Differences abound in the presence of certain plasmid replicons across different serotypes, sources, and geographic locations. The majority of IncFII and IncFIB replicons were co-carried and identified in *S*. Enteritidis and *S*. Typhimurium. IncX1 and IncA/C2 replicons were dominant in some serotypes, such as *S*. Newport and *S*. Dublin^12^. Among human sources, IncF is most often isolated in Asia and North and South America, followed by IncA/C, while European isolates carry plasmids with diverse replicons including IncI and IncH^13^.

The *spvRABCD* operon in subspecies I, located exclusively on the *Salmonella* virulence plasmid (pSV plasmid and associated with IncFII or IncFIB), is a significant virulence factor^14^. Isolates carrying the *spvRABCD* operon are often identified as increased virulence and enhanced disease severity^14^. The three genes (*spvR*, *spvB* and *spvC*) both necessary and sufficient for the *spv* virulence phenotype are found only in host-restricted and host-adapted serovars including Abortusovis, Abortusequi, Choleraesuis, Dublin, Gallinarum/Pullorum, Paratyphi C, and Sendai, as well as some invasive strains of broad-host serovars such as Enteritidis, *S*. I.4.[5].0.12:i:-, and Typhimurium^15^. Recently, the emergence of hybrid virulence-resistance plasmids in serotypes including *S*. Enteritidis^16^ have been reported and pose a potentially serious threat to public health because they could be transferred to other *Salmonella* serotypes. Moreover, the use of antimicrobial agents could serve as co-selection pressure to spread such hybrid virulence and resistance plasmids^17^.

Phylogenetic analyses based on single-nucleotide polymorphisms (SNPs) revealed extensive plasticity of *S*. Enteritidis populations^5,19,20^. Depending on the scale and scope of the study^21^, at least three to four lineages of *S*. Enteritidis have been described globally. Additionally, two distinct (sub)lineages, largely endemic to Africa, that are strongly associated with MDR and invasive disease^5^. It was also established that international trade of poultry breeding stocks could be a driving force in the geographic dispersal of *S*. Enteritidis^21^. However, after initial global spread, how individual clones have localized, expanded, and diversified is not yet fully understood.

AMR and disease severity in humans caused by *S*. Enteritidis vary greatly from country to country. Comparative genomic analysis of over 600 *S*. Enteritidis isolates from 45 countries showed that *S*. Enteritidis lineages from developed countries were strongly associated with gastroenteritis, whereas lineages from Africa were mostly associated with MDR, causing blood stream-invasive infections^5^. However, the genomic contribution to high prevalence of MDR or increased resistance to current antibiotics of last resort in *S*. Enteritidis from Asia, specifically China, has not been well studied. To further understand the phylogeny and genomic diversity of *S*. Enteritidis clinical isolates from China in relation to the rest of the world, we have sequenced and downloaded a total of 197 *S*. Enteritidis genomes, originating from China, the U.S., Europe, and Africa and have evaluated the virulome, resistome, and transmission potential of these isolates in a global context.

## Results

### Sample dataset description

To reconstruct the phylogeny of *S*. Enteritidis human isolates in a global context, 159 genomes of *S*. Enteritidis from different countries were selected and downloaded from our initial query of public data in addition to the 16 *S*. Enteritidis genomes our laboratory sequenced in the study. In order to refer to other published *S*. Enteritidis phylogenies, 22 additional genomes (from major HierBAPS clades 2, 4, 5, and 9) of *S*. Enteritidis isolates from Nicholas Feasey’s study^5^ were also downloaded from the public database, EnteroBase. Qualities of the assemblies were checked. Genomes with poor assemblies and incomplete metadata were replaced. The final data set included 197 genomes from clinical isolates collected between 2012 and 2018 except those in Feasey’s study (Table S1). A total of 50% (98/197) were isolated from North America, 31% (62/197) from Europe, 8% each (16/197, 16/197) from Africa and Asia, and 3% (5/197) from South America. The genome of *S*. Gallinarum strain 287/91, a close neighbor of *S*. Enteritidis, was included as an outgroup.

### MLST profiles

Five different MLST profiles, (ST11, ST183, ST1925, ST1974, and ST5895), were observed in the 197 genomes analyzed here (Table S1). Interestingly, 95% (187/197) of *S*. Enteritidis genomes were ST11, ST183 was represented by 5 isolates from the UK (2.5%, 5/197) in clade IV, a distant clade consisting of five UK human isolates.

### The phylogenetic structure of *Salmonella* Enteritidis

**Figure 1:**
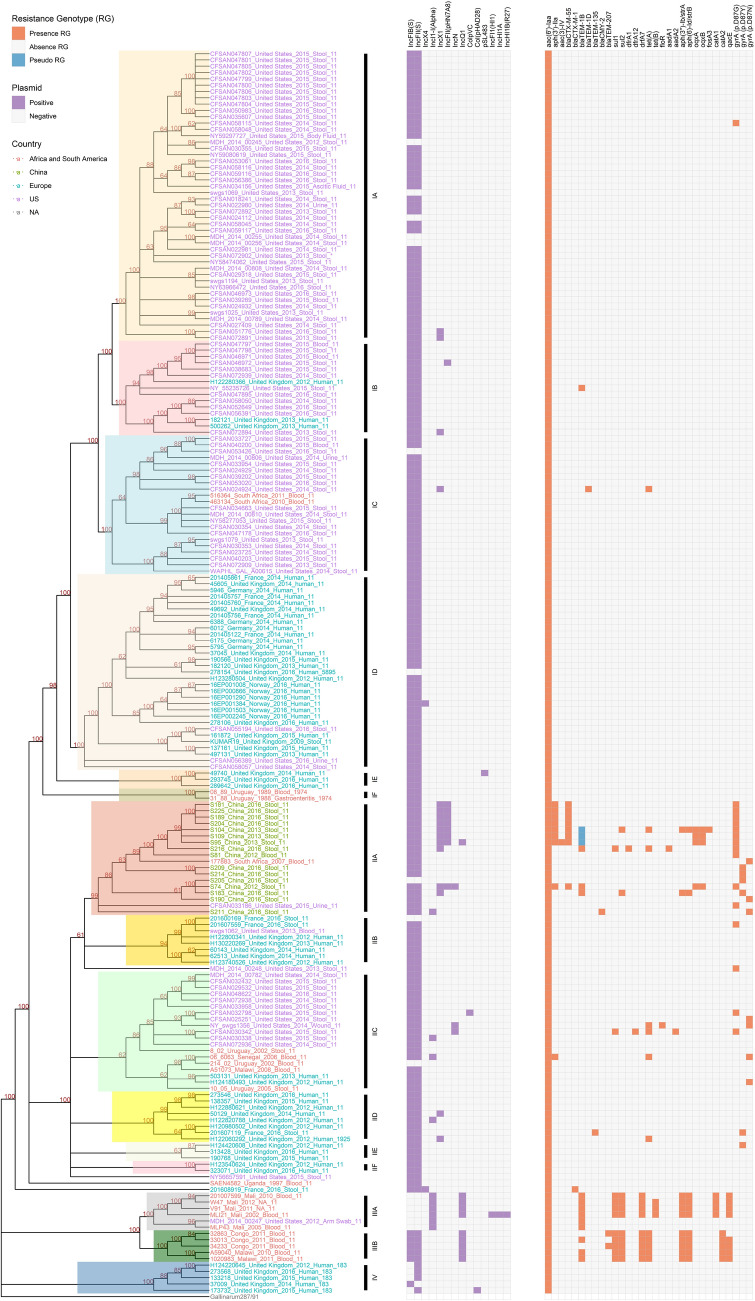
Phylogeny of *S. enterica* serovar Enteritidis isolates from humans. A phylogenetic tree is generated using the maximum likelihood method with 10,077 concatenated SNPs in the core genome sequences of 197 genomes. The root is identified using *S*. Gallinarum 287/91 as the outgroup. Bootstrap values (> 60%) are presented on the branches. Named clades are color coded in the tree. The geographical isolation source of each isolate in the tree is indicated by colored font: Africa and South America countries, red; China, green; European countries, teal; United States, purple. The presence of replicon sequence (purple box), antimicrobial resistance genes (ARGs) and ns SNPs resulting in substitutions in the QRDR of GyrA (red box), and pseudo ARG (teal box) are indicated.

A SNP matrix consisting of 10, 077 nucleotides was used to reconstruct the phylogeny of *S*. Enteritidis for 197 *S*. Enteritidis genomes and the outgroup *S*. Gallinarum 287/91 (Fig. 1). Four phylogenetically and geographically well-defined clades within *S*. Enteritidis, I–IV, were identified with SNP distance ranging from 0 (CFSAN046971_2015_US and CFSAN046972_2015_US) to 1,365 (173732_2015_UK and V91_2011_Mali) SNPs. All four clades were strongly supported, each having 100% bootstrapping support (Fig. 1). Clade III was originated from sub-Saharan Africa (sSA)^18^, containing West African clade IIIA and Central/Eastern African clade IIIB. Clade IV was strongly associated with the UK isolates, all of which were ST183. Based on their distribution within the clade and identification of major splits at the base of each clade, six distinct subclades, A-F, were identified in both clade I and clade II. Subclades IA-IC were mainly composed of isolates from the U.S. (78/83), whereas most isolates from subclades ID-IF were from European countries (31/36). Clade II was a geographically diverse and more cosmopolitan clade, containing a global set of isolates. Although the 16 isolates from China were clustered together with the cosmopolitan clade (II), these isolates formed a distinct, locally restricted subclade (IIA) within the larger group (Fig. 1) with notably strong bootstrap support. It was noteworthy that in several instances on the tree that foreign travel supports the notion that may be involved in the dissemination of *S*. Enteritidis.

### Distribution of antimicrobial resistance determinant

Overall, a total of 28 antimicrobial resistance genes (ARGs), plus point mutations in *gyrA* (D87G, D87Y, and D87N) that associated with decreased susceptibility or resistance to quinolone, were identified among the isolates (Fig. 1). Comparison of the AMR properties across the four clades identified here revealed that the majority of clade I and IV isolates displayed pan-susceptible AMR profiles, with ARGs found in only sporadically several isolates in clade I (Fig. 1). In contrast, as high as 81.8% (9/11 isolates) of isolates were MDR (at least two classes of antimicrobials) in clade III. Clade II isolates showed mixed susceptible/MDR clinical isolates (46/13), And it was noted that in clade II, 77% of MDR isolates (10/13) resided in subclade IIA from China. All the isolates from subclade IIA had a point mutation in the *gyrA* gene, but none of the isolates from sSA clade (clade III) had *gyrA* mutations. Interestingly, the most common ARGs from the Chinese isolates were *aph(3’)-IIa*, *bla*_CTX-M-55_, and *bla*_TEM-1B_, whereas *bla*_TEM-1B_, *sul1*, *sul2*, *drfA7*, *strA*, and *strB* were most identified in the African isolates. In addition, plasmid-mediated quinolone resistance (PMQR) genes *oqxA/B* and quaternary ammonium compound and disinfectant resistance gene *qacE* were only identified in Chinese isolates (25%, 4/16) and African isolates (50%, 8/16), respectively.

### Plasmid identification, distribution, and genomic structures

A total of 14 Inc groups were identified among the 197 *S*. Enteritidis isolates analyzed in this study (Fig. 1). Most isolates (176/197, 89.3%) carried both IncFIB(S) and IncFII(S) replicons, but only isolates from clade IV were found to carry one of the two replicons. IncX1 and IncFII(pHN7A8) were exclusively found in Chinese MDR isolates (subclade IIA), whereas IncQ1 was highly associated with MDR isolates from sSA (clade III). Additionally, the IncN plasmid was also detected in MDR strains from other countries. It is worth noting that IncI1-I(Alpha) only appeared in isolates from West Africa (subclade IIIA). Interestingly, MLI21, an isolate from Mali, harbored additional plasmid replicons IncFI1, IncHI1A, and IncHI1B without changing its AMR profile (*bla*_*TEM-1B*_, *sul1*, *sul2*, *defA7*, *tet(B)*, *strA*, *strB*, *catA1*, and *qacE*) which was shared with other MDR isolates from subclade IIIA.

**Figure 2:**
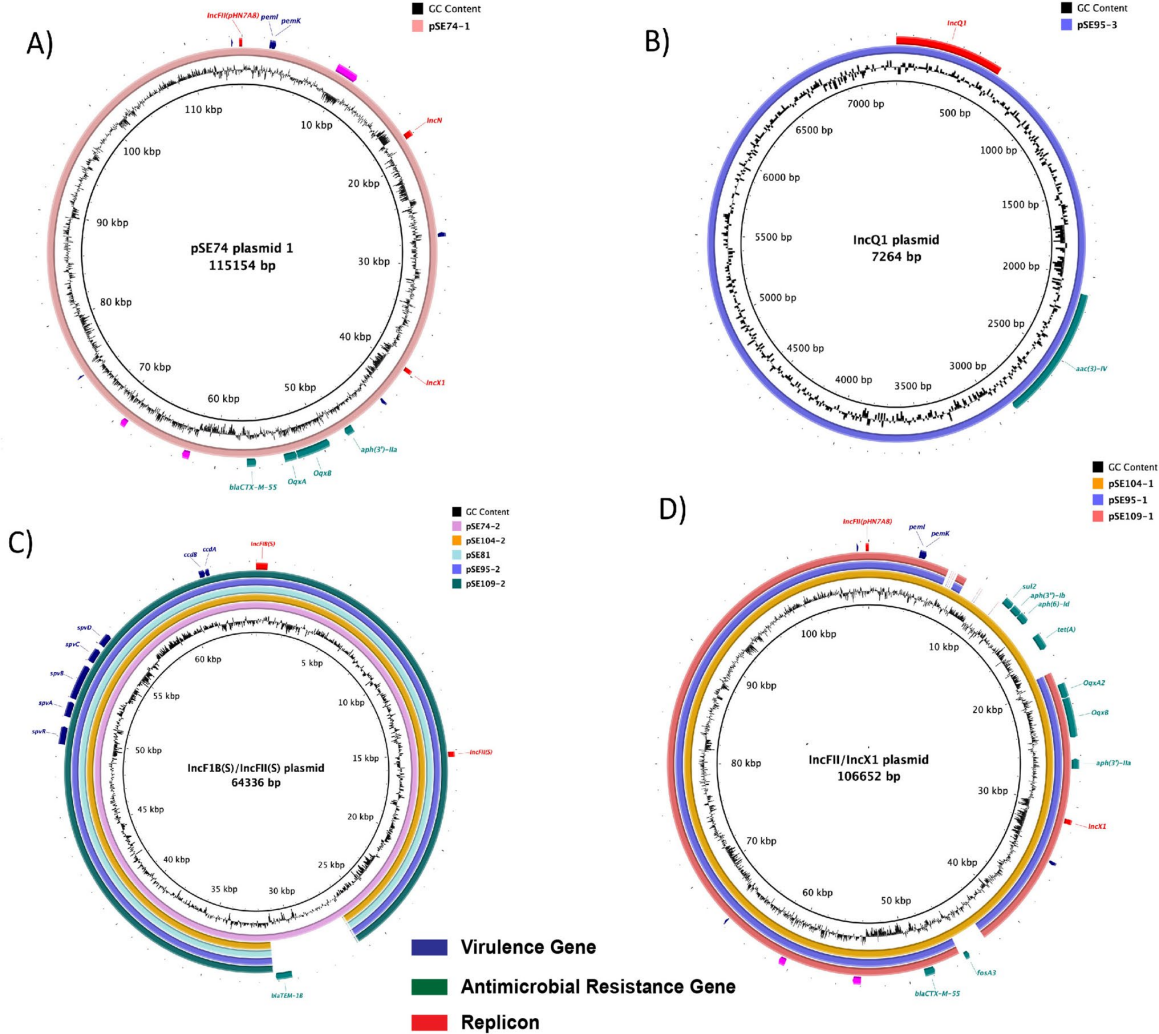
Genomic structure and comparison of the plasmids from closed genomes of five selected Chinese isolates. (**A**) IncFII(pHN7A8)-IncN-IncX1 hybrid plasmid, only identified from isolate SE74; (**B**) IncQ1 plasmid; (**C**) IncF1B(S)-IncFII(S) hybrid plasmid; (**D**) IncFII(pHN7A8)-IncX1 hybrid plasmid. Featured genes including replicons, ARGs, and virulence genes are color coded on the bottom of the figure.

Four different plasmids were identified from the closed genomes of five Chinese isolates, with each isolate carrying up to four plasmids. Three of these were hybrid plasmids (IncFII(pHN7A8)-IncN-IncX1, IncQ1, IncFIB(S)-IncFII(S), and IncFII-IncX1 plasmid), (Fig. 2). The IncFII-IncX1 hybrid plasmid carried multiple ARGs, including *sul2*, *aph(3″)-*Ib/*str*A, *aph(6)-*Id/*str*B, *tet(*A*)*, *oqx*A, *oqx*B, *aph(3’)-*IIa, *fos*A3, and *bla*_CTX-M-55_, as well as a toxin/antitoxin (T/AT) module *pem*K/I (Fig. 2D). IncFIB(S)-IncFII(S) was the most common hybrid plasmid identified, carrying the *spvRABCD* virulence operon and a T/AT module *ccd*B/A (Fig. 2C). Notably, one of the IncFIB(S)-IncFII(S) plasmids (pSE74-2) carried an ARG, *bla*_TEM-1B_, in addition to the virulence operon (Fig. 2C).

### Distribution of *Salmonella* pathogenicity islands (SPIs), SGI-1, and *spv* virulence operon

**Figure 3:**
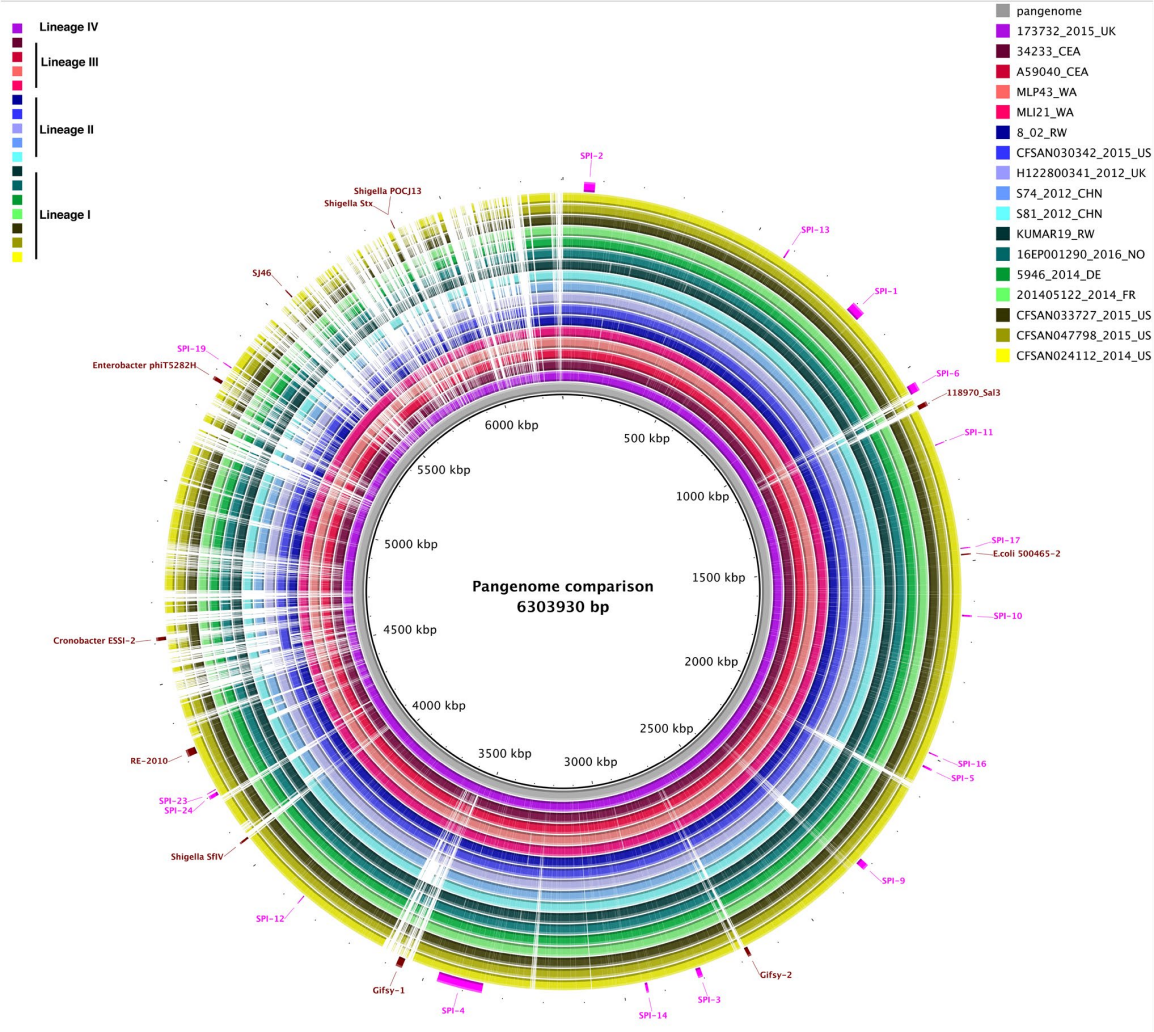
Genome comparison of selected lineage specific isolates against the pangenome generated from 197 *S*. Enteritidis genomes using Roary v3.12.0. Legend showing color gradient for % similarity. The prophage regions and *Salmonella* pathogenicity islands (SPIs) from the pangenome are annotated in Artemis and marked in brown and pink, respectively.

All isolates carried 15 SPIs, including SPIs-1 to 6, 9 to 14, 16, 17, and 19, and none of the isolates carried SPI-7, 8, 15, 18, 20, 21 and 22 (Fig. 3, Table S1). SPI-23 was completely missing from all of the isolates from sSA (clade III) but was present in the remaining 94% (185/197) of isolates, except for one isolate in clade IID from France (201,607,119). SPI-24, however, was observed in all isolates from clade III, 93% of clade I isolates (111/119), and 95% of clade II isolates (56/59), but absent entirely in clade IV. Notably, the *spv* operon was identified in 187 *S*. Enteritidis isolates (95%) and was highly associated with IncFII and IncF1B replicons (Fig. 1), except for the isolates in subclade IIIA, which did not contain either of the IncF replicons. Remarkably, no trace of SGI-1 was detected in any of the *S*. Enteritidis isolates interrogated here.

### Accessory genome

**Figure 4:**
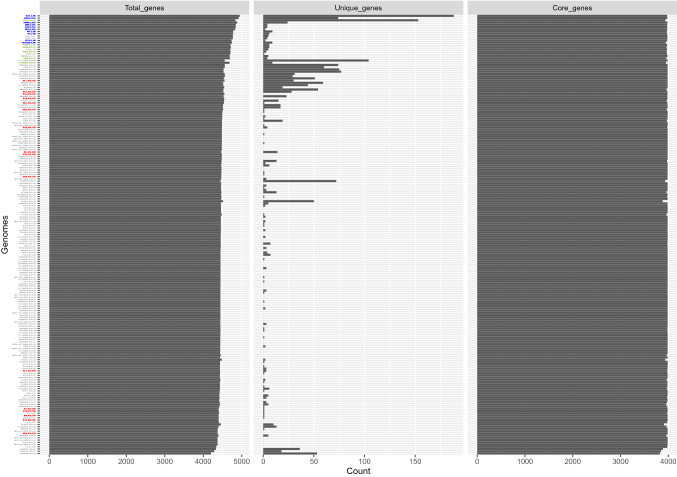
Plot of number of genes, unique genes, and core genes per genome among the 197 *S*. Enteritidis genomes.

**Figure 5:**
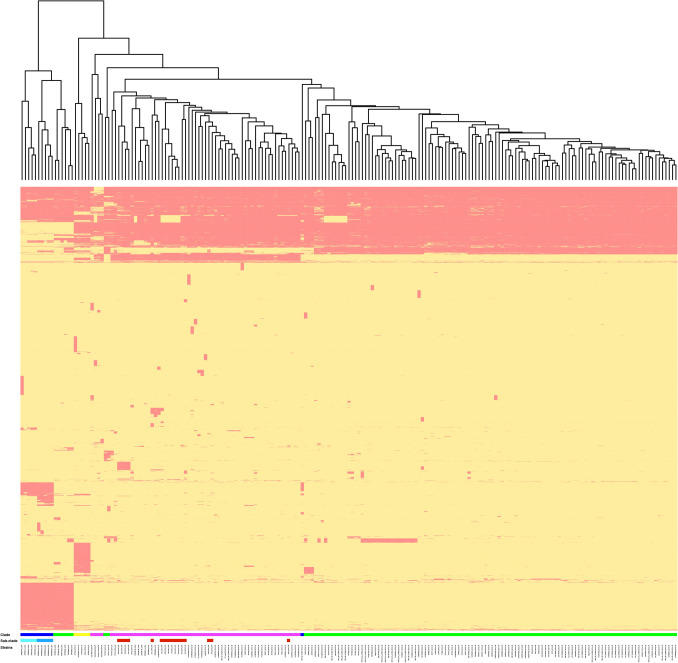
Heatmap of gene presence (red) and absence (yellow) in the accessory genomes. The cluster diagram on the top is based on the presence/absence matrix. Clade and sub-clade that each isolate belonged to in the phylogenetic tree was color coded.

A pangenome size of 8,191 nonredundant genes was created using annotated de novo assemblies of all *S*. Enteritidis isolates. The core genome of *S*. Enteritidis comprises 3653 genes (Fig. 4), and genes with a prevalence of 15% < x < 99% (shell genes) numbered 905. Additionally, genes with a prevalence of < 15% (cloud genes) numbered 3633. Based on gene presence-absence matrices, accessory genome composition appeared to be influenced considerably by geography (Fig. 5). The temperate prophages were one of the major compositions in the accessory genome, contributing hotspots for enhanced horizontal gene transfer. Prophages were nearly ubiquitous in this *S*. Enteritidis collection save for isolate 50,129 from the UK. In total, PHASTER identified 37 intact prophages among 197 genomes with each *S*. Enteritidis isolate containing 1–8 different intact prophages from other bacterial species (Table S3). The five most common prophages identified in *S*. Enteritidis isolates were Gifsy-2 (183/197, 92.9%), *Escherichia coli* phage 500,465–2 (173/197, 87.8%), *Salmonella* phage SJ46 (164/197, 83.2%), *Shigella* phage SflV (148/197, 75.1%), and *Salmonella* phage RE-2010 (114/197, 57.9%) (Table S3). Some phylogenetic difference in prophage distribution was noted (Fig. 3). That is, RE-2010 was only found in clade I, and was entirely absent from the other clades. Prophage 118,970-sal3 was abundant in clades II and IV, but very rare in clades I and III. While Gifsy-1 was abundant (100%) in clade III but was identified in a very low percentage (6/181, 3.3%) in clades I and II. Interestingly, Gifsy-2 was opposite in its distribution, and isolates from clade IV contained both Gifsy-1 and Gifsy-2. Finally, Enterobacter phage phiT5282H was only found in clade III.

Accessory genomic features between the *S*. Enteritidis clades were distinct. Identification of clade II specific genes was defined as: abs (clades II_prevalence – clades I/III/IV_prevalence) > 0.99. Using the criteria previously mentioned as well as an in-house R script, 93 genes were identified to be clade II specific, with 57 genes present in all of the isolates in clade II (Table S2). Gene-ontology enrichment analysis revealed that genes involved in cellular and metabolic processes were highly enriched. For example, isolates in clade II harbored multiple genes responding to a variety of stimuli including toxins, pH, antibiotics, and other abiotic stressors.

## Discussion

AMR and disease severity caused by *S*. Enteritidis vary greatly from different regions^23–25^. This study has attempted to understand the genomic features associated with virulence, resistance, and geographical origin of *S*. Enteritidis on a global level.

In the study, geographical structure is documented within our *S.* Enteritidis phylogeny and sub-phylogenies. For example, clade III within sSA^18^ and clade IV within the U.K. are detected in the *S*. Enteritidis population. Geographical sub-clustering is also evident within clades I and II. While clade I is composed mainly of human isolates from the U.S. and Europe, including some of the isolates from Germany during the 2014 European *S*. Enteritidis outbreak^26^, clade II displays a much greater global cosmopolitan geographic diversity. To be able to make comparisons to other reported phylogenies^5,21^, clade specific human isolates from a West African clade, a Central/East African clade, an outlier clade, and a global epidemic clade in the study of Feasey et al.^5^ were used in the construction of the phylogeny presented in this study. In agreement with previous observations^5,21^, four well-supported clades were found in this study, including clade III (A and B) (*i.e.* sSA clade), clade I (*i.e.* outlier or Atlantic clade) and clade II (*i.e.* global/cosmopolitan clade). All of the isolates from China formed a subclade within clade II, the global epidemic clade, suggesting that the established clone of *S*. Enteritidis in China has persisted and evolved remotely within this specific geographical region, much like the localization of African epidemic lineages within the sSA area. Similar geographical partitions were reported in other *Salmonella* serovars in a global context^27–29^. These data again suggest that besides anthropogenic factors, the evolution of *S. enterica* genomes is also strongly associated with ecological preference and geographical distribution. Notably, one U.S. isolate (MDH_2014_00247 from arm swab) belonged to the sSA clade, and one U.S. isolate (CFSAN033186 from urine) and one South African isolate (177,883 from blood) fell within the Chinese subclade. These isolates were likely associated with international travels to these areas^5,30^. However, detailed travel history or other epidemiological information will be needed for further inferences.

The most frequent profile within the collection was ST11 (95%) followed by ST183. ST11 represents the most prevalent MLST profile among *S*. Enteritidis worldwide (94.8%)^22^. In EnteroBase, there were 1150 released *S*. Enteritidis genomes were identified as ST183 as of Mar. 1, 2021. The isolates were mainly isolated from the UK, France, and Germany. ST183 is an emerging and endemic sequence type isolated from hedgehogs in western Europe and human in the UK and appears more likely to infect children aged four or younger^31^. In addition, a recent study reported that isolates of ST183 were the only isolates associated with hedgehogs in Germany^32^.

Antibiotic resistant *Salmonella* infections have been linked to severe clinical outcomes including bloodstream infections, meningitis, septicemia, and high hospitalization and fatality rates^23,33^. A high proportion of invasive non-typhoidal *Salmonella* (iNTS) isolates have been shown to be MDR^5,8^. Human isolates from this study that fall in the China subclade as well as in the sSA clade showed the highest abundance of resistance genes and presence of extended spectrum beta-lactamase (ESBL) genes. However, the pattern of most common resistance genes was different between the two geographic locations. Isolates from the sSA clade (clade III) exhibited a MDR genotype, with associated genes as *bla*_TEM-1B_, *sul*, *dfrA*, *str*, and *cat*. Isolates from the Chinese subclade, however, displayed a much more complex AMR patterns. Some isolates (3/16) had a similar MDR genotype as isolates from the sSA clade showed. Notably, clade II (global/cosmopolitan clade) has a much higher percentage of isolates (47.4%, 28/59) including all 16 isolates from China that contained mutations in quinolone resistance determining regions (QRDR), such as *gyrA* mutations, when compared to other clades (0.8% in clade I; 0% in clades III and IV). Moreover, only isolates from China harbored the plasmid-mediated quinolone resistance (PMQR) genes *oqxA/B*. These findings may be explained by a gradual decline in the percentage of MDR isolates accompanied by a steady increase in the rates of *Salmonella* isolates resistance to nalidixic acid, cefotaxime, and ciprofloxacin during 2007–2016. The ciprofloxacin and cefotaxime resistance rates in *S*. Enteritidis in China have been of great concern as they are much higher than those in the U.S. and Africa^34^. In addition, the China subclade carried several beta-lactamase genes of significant public health concern, such as ESBL and AmpC-like-lactamases, including *bla*_CTX-M-55_, *bla*_TEM-1B_, and *bla*_CMY-2_. Notably, these genes were also reported as the most common ESBL genes carried in the ceftriaxone-resistant isolates in China during 2007–2016^8^.

The ARGs seen here were mainly acquired and plasmid-borne except the non-functional *aac(6’)-Iaa* gene, located on the chromosomes of all the *S*. Enteritidis genomes. A clade-specific Inc group was also observed in this study. It is noteworthy that IncFII(pHN7A8) was predominantly associated with genotypic MDR profiles found in *S*. Enteritidis clinical isolates from China, while AMR determinants in isolates from sSA (clade III) were mainly linked to the IncQ1 replicon type. Among plasmids identified in five isolates from China, both IncFII(pHN7A8)-IncX1 and IncFII(pHN7A8)-IncN-IncX1 type hybrid plasmids acquired multiple ARGs^35^. In addition, both plasmids had a complete conjugative system: the *oriT*, the relaxase, the type IV coupling protein (T4CP), and the type IV secretion system (TIVSS), together with 22 *tra* genes belonging to F conjugative system, confirming the potential capacity of these plasmids to be transferred to other bacteria via conjugation. Interestingly, both plasmids possessed the typical IncFII(pHN7A8) backbones from pHN7A8, a chimeric multidrug resistance plasmid from *E. coli* of animal originated from China^36^. Due to the abundant presence of IS26, a mosaic structure of the MDR region was observed in IncFII(pHN7A8)-like plasmids detected in this study, suggesting evolution through the recombination and integration of a variety of ARGs. All of the sequenced IncFII(pHN7A8)-like plasmids had acquired the *oqx*A, *oqx*B, *aph(3’)-*IIa, and *bla*_CTX-M-55_ genes, while pSE104-1 acquired additional genes (*sul2*, *aph(3″)-*Ib/*str*A, *aph(6)-*Id/*str*B, *tet(*A*)*, and *fosA3*). These plasmids also carried the addiction system *pemI*/*pemK*, which is involved in stable inheritance of the plasmid. Notably, the pHN7A8-like plasmid has been disseminated to multiple species in the *Enterobacteriaceae* family^37^ but was predominantly confined geographically. All the plasmids with the same pHN7A8 origin of replication were isolated in Asia, especially China^38^. Recently, two pHN7A8-like plasmids were detected in clinical isolates of *K. pneumonia*e from Bolivia^39^ and *E. coli* from a wastewater treatment plant (WWTP) from Barcelona, Spain^40^, respectively, with most variation associated with ARGs, demonstrating a possible intercontinental dissemination. In the U. S., pHN7A8 was detected in *E. coli* isolated from a forest in North Carolina without carrying any ARGs. A similar IncFII(pHN7A8) without ARGs was detected here in one clinical isolate (CFSAN046972) of *S*. Enteritidis from the U.S. Among the nine isolates from China carrying the *bla*_CTX-M-55_ gene, 89% (8/9) of them was strongly associated with the IncFII(pHN7A8) replicon. In a most recent study, fosfomycin and ceftriaxone co-resistance profiles were found in 2.8% (14/501) of clinical *S*. Enteritidis isolates in China^41^. It was noted that most (64.3%, 9/14) *bla*_CTX-M-55_ and *fosA3-*bearing plasmids possessed a typical IncFII backbone similar to the plasmid pHN7A8 carrying *bla*_CTX-M-55/-65_, *fosA3*, and *rmtB* resistance genes from an *E. coli* strain (67% coverage, 99.94% identity). Co-existence of the *bla*_CTX-M-55_ and *fosA3* genes was also found in one of the Chinese clinical isolates (S104) in this study. All together, these findings illustrate the role of horizontal gene transfer of IncFII(pHN7A8)-like plasmids in the dissemination of ARGs in *S*. Enteritidis in China^41^.

The prevalence of the *spv* operon in *S*. Enteritidis isolates can vary from 15.1 to 93.5% depending on the geographic location^42,43^. It was also noted that a lower percentage of strains from poultry carried a *spv* loci than that from humans^44^. In this study, 94.9% of *S*. Enteritidis isolates (n = 187) were found to have the *spvRABCD* operon. The *spv* operon was located on a hybrid plasmid IncFIB(S)-IncFII(S) in five closed genomes from China. The hybrid plasmids from these isolates are around 60–64 kb, comprising serovar specific features^45^. In addition to the *spv* operon, the plasmid carried other virulence loci. For example, the *pef* region mediates adhesion to intestinal epithelial cells for various species^14^. Also, YjiK is linked to quorum sensing, which may reduce sensitivity to ceftiofur^46^. The plasmid also carried the addiction system *ccdA*/*ccdB.* The *spv* operon was found in a variety of virulence plasmids, including IncX1, IncFII, IncA/C-IncFI1, IncA/C-IncFII^12,47^. In this study, the prevalence of the *spv* operon was strongly correlated with the presence of IncFIB(S)/IncFII(S) plasmid. It is also noted that the IncFIB(S)-IncFII(S) virulence plasmid SE74 possessed a laterally acquired resistance gene *bla*_TEM-1B_. The *bla*_TEM-1B_ gene was associated with Tn3 and lies adjacent to a Y-family DNA polymerase, which encodes a DNA polymerase specialized in translesion synthesis. Unlike in pUO-SeVR1, the *tra* locus found on the common virulence plasmid in *S*. Enteritidis, has undergone a certain amount of evolutionary degradation leading to the loss of mobilization of the virulence plasmid^48^. Hence, the transmission of this new type of virulence plasmid may be only vertical within *Salmonella* serovars^45^. In theory, plasmids, belonging to the same Inc group are not able to stably replicate in the same bacterial cell. However, the emergence of hybrid plasmids, has complicated this incompatibility. Notably, several clinical isolates from China possessed both a virulence plasmid and a resistance plasmid, presenting a new challenge in treating *Salmonella* infections caused by *S*. Enteritidis^41^.

Clustering of the ancillary genes between geographic regions was observed based solely upon presence-absence matrices. Representing a large part of the *S. enterica* accessory genome, SPIs are mostly serovar specific^18^. The absence of SPI-23 or SPI-24 in this collection of *S*. Enteritidis genomes, however, was found largely to be clade and geographic specific. SPI-23, for instance, carrying genes encoding another set of type III effector proteins, was shown to contribute to *Salmonella* invasion of porcine tissue, for example, the jejunum^49^. The loss of SPI-23 in the clade III genomes, originating from the sSA area, may provide improved fitness to these isolates in human infections during epidemics in Africa. Prophages, another major source of accessory genes, contribute to genome evolution and provide additional functions to the bacterial host via integration of virulence genes and ecologically significant genes^50,51^. Clade-specific prophage distribution patterns observed in this study are intriguing and may be used as evolutionary biomarkers. Nonetheless, further studies are needed to investigate the role of prophages in *S*. Enteritidis evolution and ecological fitness.

In summary, the current study presents an in depth phylogenetic and genomic analyses of a global collection of clinical *S*. Enteritidis genomes. Analysis of accessory genome content including AMR genes, plasmids, prophage regions, and SPIs, all provided evidence of a significant degree of geographical structuring within the *S*. Enteritidis phylogeny and its sub-phylogenies. These data suggested the persistence and continued parallel evolution of *S*. Enteritidis within a specific geographical region such as China and sSA after the clone was introduced and established. Future phylogenetic studies including more clinical *S*. Enteritidis genomes from Asiatic countries and Oceanic countries will help to shed light on *S*. Enteritidis evolution and its global AMR distribution and dissemination.

## Materials and methods

### Bacterial strains

A total of 16 *S.* Enteritidis clinical isolates collected from China isolated between 2012 and 2016, were used in this study. All isolates were stored in tryptic soy broth (TSB; Fisher Scientific Inc., Hampton, NH) at − 80 °C with 20% glycerol until use. In addition, whole genome sequence (WGS) data of 181 human clinical *S*. Enteritidis isolates from 12 other countries, including the U.S. (n = 98), the UK (n = 42), Germany (n = 5), France (n = 9), Norway (n = 6), Mali (n = 5), Malawi (n = 3), Congo (n = 3), Uganda (n = 1), South Africa (n = 3), Senegal (n = 1), and Uruguay (n = 5), were downloaded from the National Center for Biotechnology Information (NCBI) Pathogen Detection (https://www.ncbi.nlm.nih.gov/pathogens/) website and database for comparative genomic analysis (Table S1). The selection criteria of isolates from the U.S., UK, Germany, France, and Norway are based widely on the time period of isolation, the quality and availability of complete annotated genomes and their associated metadata and does not necessarily representative of a specific collection of strains within an individual country. In addition, the criteria of isolates from Africa and Uruguay was also based on phylogenetic analysis from an earlier study^5^.

### Genome sequencing, assembly and annotation

Genomic DNA was extracted using the DNAeasy Blood and Tissue Kit (Qiagen, Inc., Valencia, CA) following an overnight grown culture incubated at 37 °C in TSB (Fisher Scientific Inc., Hampton, NH). After measuring DNA concentrations with a Qubit 3.0 fluorometer (Life Technologies, MD), libraries were prepared following the Illumina Nextera XT DNA Library Preparation (Illumina, San Diego, CA). The prepared libraries were sequenced on the Illumina MiSeq platform (Illumina, San Diego, CA) using the MiSeq Reagents Kits v2 (500 cycles) or MiSeq Reagents Kits v3 (600 cycles) with paired end option. Raw reads quality was assessed by the following parameters: cluster density (1200–1400 k/mm^2^) and percentage of clusters passing filters (> 80%). Raw reads were trimmed with Trimmomatic^52^ and de novo assembled using SKESA v2.2 with minimal contig length of 500 bp^53^. Based on the AMR profiles, five isolates (S74, S81, S95, S104, and S109) were selected to close the genomes using the Nanopore sequencing technology as previously described^54^. Genomes were annotated using Prokka v1.14.5^55^.

### Detection of SPIs and *spvRABCD* virulence operon

To evaluate the virulence potential of all *S*. Enteritidis virulence factors, all SPIs and *spvRABCD* virulence operon were examined. An in-house bioinformatic pipeline^12^ was constructed to identify 24 SPIs. The existence of SPIs were identified by BLASTn searches of the assembled genomes against a local database. Sequence of the *spvRABCD* virulence operon from *S*. Dublin plasmid pOU1115 (Accession: DQ115388) was used for a local BLASTn search (e-value: 1e^−3^) to identify its presence/absence in all 197 *S*. Enteritidis isolates.

### Identification of AMR genotypes, chromosomal point mutations, plasmid types, and *Salmonella* genomic island-1 (SGI-1)

To assess AMR genotype profiles, ResFinder 4.1^56,57^ (last updated on May 27, 2021) was used for detection of both acquired AMR genes and chromosomal point mutations that associated with specific resistance phenotypes using the ResFinder default settings (90% minimum identity and 60% minimum sequence length coverage for nucleotide sequence). In addition, PlasmidFinder 2.1^58^ (last updated on July 1, 2020) was used to determine the plasmid type with default settings (95% sequence identity and 60% sequence length coverage for nucleotide sequence). A heatmap illustrating the presence/absence of AMR genes, chromosomal point mutations, and plasmid types was created. The BLAST Ring Image Generator (BRIG) v0.95^59^ was also used for comparison analysis of the plasmids in the five closed genomes of *S*. Enteritidis from China and for image generation. The presence of the SGI-1, an integrative mobilizable element carrying a cluster of resistance genes, was detected by blasting genomes against the backbone of SGI based on the SGI structure reported by Hall^60^ using an in-house bioinformatics pipeline^12^.

### Whole-genome phylogenetic analysis and multi-locus sequence typing (MLST)

To elucidate evolutionary relationships amongst *S*. Enteritidis isolates, whole-genome phylogenies were reconstructed using *S*. Gallinarum 287/91 as an outgroup. Briefly, Fastq data from Illumina raw reads of 197 *S*. Enteritidis isolates were used as inputs for SKESA v2.2 to generate assemblies in fasta file format with minimal contig length of 500 bp. kSNP3.0^61^ with optimum k value as 19 from Kchooser was used to generate a matrix of core SNPs. GARLI 2.01^62^ was used to construct the maximum likelihood phylogenetic tree (ratematrix = 6rate, ratehetmodel = gamma). Multiple runs were performed (n = 100) to ensure that results were consistent. To estimate support for each node, phylogenies were reconstructed for 1,000 bootstrap replicates of the data set. Python program SumTrees v4.0.0^63^ was used to generate one consensus tree with bootstrap value at a 60% threshold, and ggtree v3.1.3^64^ was used to visualize the phylogenetic tree. Circular BLAST comparison of the representative draft whole genome sequences from each major clade was performed using BRIG v0.95. In addition, EnteroBase^33^ (https://enterobase.warwick.ac.uk/species/index/senterica) was used to determine the ST using seven housekeeping genes (*aroC*, *dnaN*, *hemD*, *hisD*, *thrA*, *sucA*, and *purE*) for studied *S*. Enteritidis isolates, and MEGA7^65^ was used to estimate the evolutionary divergence between sequences and to generate the pairwise distance matrix.

### Pan-genome analysis

To identify clade specific genes, all assemblies in Fasta file format generated from SKESA v2.2 were annotated with Prokka 1.14.5 to generate GFF3 files as the inputs for pan-genome calculation using Roary v3.12.0^66^. Those genes present in at least 99% of the isolates were considered core genome and genes present in 100% of the isolates in the clade were considered as clade specific genes. The matrix with the presence/absence of core and accessory genes was then used as input for an in-house R script to generate heatmap. The Clusters of Orthologous Groups of proteins (COGs) database was used for functional annotation. At least one GO (Gene Ontology) term was assigned to all clade specific genes for functional enrichment analysis.

### Identification and in silico characterization of prophage sequences

Potential prophage profiling among 197 *S*. Enteritidis genomes were detected and annotated by PHASTER (Phage Search Tool Enhanced Release)^67^. According to the completeness of identified prophage regions in the bacterial genome, PHASTER classifies those regions into three classes including intact, incomplete and questionable on the basis of the score obtained (*i.e.* score > 90, score 70–90, and score < 70, respectively).

## Supplementary Information

Supplementary Information.

## Data Availability

All 16 *S*. Enteritidis Chinese human isolates are available in the NCBI database with the specific SRA accession and Genome accession are listed in Supplementary Table 1. The SRA accession numbers for the rest genomes from the collection are showed in Supplementary Table 1 as well.
