# Supplementary material for: Geography shapes the genomics and antimicrobial resistance of Salmonella enterica Serovar Enteritidis isolated from humans
Source: Sci Rep. 2023 Jan 24;13:1331. doi: 10.1038/s41598-022-24150-4 (PMC9873609; doi:10.1038/s41598-022-24150-4)
Supplement: Supplementary file 1 — Supplementary Information. [file 41598_2022_24150_MOESM1_ESM.pdf]

Geography Shapes the Genomics and Antimicrobial Resistance of *Salmonella enterica* Serovar Enteritidis Isolated from Humans

Guojie Cao[1*], Shaohua Zhao[2], Dai Kuang[3], Chih-Hao Hsu[2], Lanlan Yin[1], Yan Luo[1], Zhao Chen[4], Xuebin Xu[5], Errol Strain[2], Patrick McDermott[2], Marc Allard[1], Eric Brown[1], Jianghong Meng[4], Jie Zheng[1*]

[1]Center for Food Safety and Applied Nutrition, U.S. Food and Drug Administration, College Park, MD
[2]Center for Veterinary Medicine, U.S. Food and Drug Administration, Laurel, MD
[3]Ruijin Hospital, School of Medicine, Shanghai Jiao Tong University, Shanghai, China
[4]Joint Institute for Food Safety and Applied Nutrition, Center for Food Safety & Security Systems, Department of Nutrition and Food Science, University of Maryland, College Park, MD
[5] Shanghai Municipal Center for Disease Control and Prevention, Shanghai, China

*Co-Corresponding Authors:
Jie Zheng, Jie.Zheng@fda.hhs.gov
Guojie Cao, Guojie.Cao@fda.hhs.gov

Table S1. *Salmonella* Enteritidis human isolates in the collection and associated genomic information on *Salmonella* Pathogenicity Islands (SPIs), presence of *spv* genes and sequence type (ST).

Isolate	Country	Year	Source	RA Accession No	WGS Accession No.	SPIs	spv	ST
S74	China	2012	Stool	SRR11425857	CP050723	SPI1-6, SPI9-14, SPI16-17, SPI19, SPI23, SPI24	positive	11
S81	China	2012	Blood	SRR11425854	NZ_CP050721	SPI1-6, SPI9-14, SPI16-17, SPI19, SPI23, SPI24	positive	11
S95	China	2013	Stool	SRR11425852	NZ_CP050716	SPI1-6, SPI9-14, SPI16-17, SPI19, SPI23, SPI24	positive	11
S104	China	2013	Stool	SRR11425850	CP050712	SPI1-6, SPI9-14, SPI16-17, SPI19, SPI23, SPI24	positive	11
S109	China	2013	Stool	SRR11425848	NZ_CP050709	SPI1-6, SPI9-14, SPI16-17, SPI19, SPI23, SPI24	positive	11
S181	China	2016	Stool	SRR14574475	JAHIBE000000000	SPI1-6, SPI9-14, SPI16-17, SPI19, SPI23, SPI24	positive	11
S183	China	2016	Stool	SRR14574474	JAHIBD000000000	SPI1-6, SPI9-14, SPI16-17, SPI19, SPI23, SPI24	positive	11
S189	China	2016	Stool	SRR14574472	JAHIBC000000000	SPI1-6, SPI9-14, SPI16-17, SPI19, SPI23, SPI24	positive	11
S190	China	2016	Stool	SRR14574471	JAHIBB000000000	SPI1-6, SPI9-14, SPI16-17, SPI19, SPI23, SPI24	positive	11
S204	China	2016	Stool	SRR14574470	JAHIBA000000000	SPI1-6, SPI9-14, SPI16-17, SPI19, SPI23, SPI24	positive	11
S205	China	2016	Stool	SRR14574469	JAHIAZ000000000	SPI1-6, SPI9-14, SPI16-17, SPI19, SPI23, SPI24	negative	11
S209	China	2016	Stool	SRR14574468	JAHIAY000000000	SPI1-6, SPI9-14, SPI16-17, SPI19, SPI23, SPI24	positive	11
S211	China	2016	Stool	SRR14574467	JAHIAX000000000	SPI1-6, SPI9-14, SPI16-17, SPI19, SPI23, SPI24	positive	11
S214	China	2016	Stool	SRR14574466	JAHIAW000000000	SPI1-6, SPI9-14, SPI16-17, SPI19, SPI23, SPI24	positive	11
S216	China	2016	Stool	SRR14574465	JAHIAV000000000	SPI1-6, SPI9-14, SPI16-17, SPI19, SPI23, SPI24	positive	11
S225	China	2016	Stool	SRR14574473	JAHIAU000000000	SPI1-6, SPI9-14, SPI16-17, SPI19, SPI23, SPI24	positive	11
5795	Germany	2014	Human	SRR3410212	NA	SPI1-6, SPI9-14, SPI16-17, SPI19, SPI23, SPI24	positive	11
5946	Germany	2014	Human	SRR3410213	NA	SPI1-6, SPI9-14, SPI16-17, SPI19, SPI23, SPI24	positive	11
6012	Germany	2014	Human	SRR3410214	NA	SPI1-6, SPI9-14, SPI16-17, SPI19, SPI23, SPI24	positive	11
6175	Germany	2014	Human	SRR3410215	NA	SPI1-6, SPI9-14, SPI16-17, SPI19, SPI23, SPI24	positive	11
6388	Germany	2014	Human	SRR3410216	NA	SPI1-6, SPI9-14, SPI16-17, SPI19, SPI23, SPI24	positive	11
201405122	France	2014	Human	SRR3410217	NA	SPI1-6, SPI9-14, SPI16-17, SPI19, SPI23, SPI24	positive	11
201405756	France	2014	Human	SRR3410218	NA	SPI1-6, SPI9-14, SPI16-17, SPI19, SPI23, SPI24	positive	11
201405757	France	2014	Human	SRR3410219	NA	SPI1-6, SPI9-14, SPI16-17, SPI19, SPI23, SPI24	positive	11

201405760	France	2014	Human	SRR3410220	NA	SPI1-6, SPI9-14, SPI16-17, SPI19, SPI23, SPI24	positive	11
201405861	France	2014	Human	SRR3410221	NA	SPI1-6, SPI9-14, SPI16-17, SPI19, SPI23, SPI24	positive	11
201600169	France	2016	Stool	SRR10674503	NA	SPI1-6, SPI9-14, SPI16-17, SPI19, SPI23, SPI24	negative	11
201607119	France	2016	Stool	SRR10674505	NA	SPI1-6, SPI9-14, SPI16-17, SPI19, SPI24	positive	11
201607559	France	2016	Stool	SRR10674513	NA	SPI1-6, SPI9-14, SPI16-17, SPI19, SPI23, SPI24	positive	11
201608919	France	2016	Stool	SRR10674517	NA	SPI1-6, SPI9-14, SPI16-17, SPI19, SPI23, SPI24	positive	11
16EP000866	Norway	2016	Human	ERR2676735	NA	SPI1-6, SPI9-14, SPI16-17, SPI19, SPI23, SPI24	positive	11
16EP001008	Norway	2016	Human	ERR2676733	NA	SPI1-6, SPI9-14, SPI16-17, SPI19, SPI23, SPI24	positive	11
16EP001290	Norway	2016	Human	ERR2676731	NA	SPI1-6, SPI9-14, SPI16-17, SPI19, SPI23, SPI24	positive	11
16EP001384	Norway	2016	Human	ERR2676732	NA	SPI1-6, SPI9-14, SPI16-17, SPI19, SPI23, SPI24	positive	11
16EP001503	Norway	2016	Human	ERR2676736	NA	SPI1-6, SPI9-14, SPI16-17, SPI19, SPI23	positive	11
16EP002245	Norway	2016	Human	ERR2676734	NA	SPI1-6, SPI9-14, SPI16-17, SPI19, SPI23, SPI24	positive	11
133218	nited Kingdoı	2015	Human	SRR5757182	NA	SPI1-6, SPI9-14, SPI16-17, SPI19, SPI23	positive	183
137161	nited Kingdoı	2015	Human	SRR7297911	NA	SPI1-6, SPI9-14, SPI16-17, SPI19, SPI23, SPI24	positive	11
138357	nited Kingdoı	2015	Human	SRR5194051	NA	SPI1-6, SPI9-14, SPI16-17, SPI19, SPI23, SPI24	positive	11
161872	nited Kingdoı	2015	Human	SRR3312356	NA	SPI1-6, SPI9-14, SPI16-17, SPI19, SPI23, SPI24	positive	11
173732	nited Kingdoı	2015	Human	SRR5757195	NA	SPI1-6, SPI9-14, SPI16-17, SPI19, SPI23	positive	183
182120	nited Kingdoı	2013	Human	SRR5219334	NA	SPI1-6, SPI9-14, SPI16-17, SPI19, SPI23, SPI24	positive	11
182121	nited Kingdoı	2013	Human	SRR5219436	NA	SPI1-6, SPI9-14, SPI16-17, SPI19, SPI23, SPI24	positive	11
190566	nited Kingdoı	2015	Human	SRR7292873	NA	SPI1-6, SPI9-14, SPI16-17, SPI19, SPI23, SPI24	positive	11
190768	nited Kingdoı	2015	Human	SRR5220934	NA	SPI1-6, SPI9-14, SPI16-17, SPI19, SPI23, SPI24	positive	11
273546	nited Kingdoı	2016	Human	SRR5215642	NA	SPI1-6, SPI9-14, SPI16-17, SPI19, SPI23, SPI24	positive	11
273568	nited Kingdoı	2016	Human	SRR5216571	NA	SPI1-6, SPI9-14, SPI16-17, SPI19, SPI23	positive	183
278106	nited Kingdoı	2016	Human	SRR6900182	NA	SPI1-6, SPI9-14, SPI16-17, SPI19, SPI23, SPI24	positive	11
278154	nited Kingdoı	2016	Human	SRR8667281	NA	SPI1-6, SPI9-14, SPI16-17, SPI19, SPI23, SPI24	negative	5895
289642	nited Kingdoı	2016	Human	SRR5220223	NA	SPI1-6, SPI9-14, SPI16-17, SPI19, SPI23, SPI24	positive	11
293745	nited Kingdoı	2016	Human	SRR5216469	NA	SPI1-6, SPI9-14, SPI16-17, SPI19, SPI23, SPI24	positive	11
313428	nited Kingdoı	2016	Human	SRR5193621	NA	SPI1-6, SPI9-14, SPI16-17, SPI19, SPI23, SPI24	positive	11

323071	nited Kingdor	2016	Human	SRR6920101	NA	SPI1-6, SPI9-14, SPI16-17, SPI19, SPI23, SPI24	positive	11
37009	nited Kingdor	2014	Human	SRR1966925	NA	SPI1-6, SPI9-14, SPI16-17, SPI19, SPI23	positive	183
37045	nited Kingdor	2014	Human	SRR3417493	NA	SPI1-6, SPI9-14, SPI16-17, SPI19, SPI23, SPI24	positive	11
45605	nited Kingdor	2014	human	SRR3417496	NA	SPI1-6, SPI9-14, SPI16-17, SPI19, SPI23, SPI24	positive	11
49692	nited Kingdor	2014	Human	SRR3417498	NA	SPI1-6, SPI9-14, SPI16-17, SPI19, SPI23, SPI24	positive	11
497131	nited Kingdor	2013	Human	SRR7451161	NA	SPI1-6, SPI9-14, SPI16-17, SPI19, SPI23, SPI24	positive	11
49740	nited Kingdor	2014	Human	SRR1958537	NA	SPI1-6, SPI9-14, SPI16-17, SPI19, SPI23, SPI24	positive	11
500262	nited Kingdor	2013	Human	SRR7284424	NA	SPI1-6, SPI9-14, SPI16-17, SPI19, SPI23, SPI24	positive	11
50129	nited Kingdor	2014	Human	SRR1957893	NA	SPI1-6, SPI9-14, SPI16-17, SPI19, SPI23	positive	11
503131	nited Kingdor	2013	Human	SRR7902691	NA	SPI1-6, SPI9-14, SPI16-17, SPI19, SPI23, SPI24	positive	11
60143	nited Kingdor	2014	Human	SRR1966064	NA	SPI1-6, SPI9-14, SPI16-17, SPI19, SPI23, SPI24	positive	11
62513	nited Kingdor	2014	Human	SRR1958526	NA	SPI1-6, SPI9-14, SPI16-17, SPI19, SPI23, SPI24	positive	11
H120980502	nited Kingdor	2012	Human	SRR1645250	NA	SPI1-6, SPI9-14, SPI16-17, SPI19, SPI23, SPI24	positive	11
H122060292	nited Kingdor	2012	Human	SRR1639015	NA	SPI1-6, SPI9-14, SPI16-17, SPI19, SPI23, SPI24	positive	1925
H122280366	nited Kingdor	2012	Human	SRR1645898	NA	SPI1-6, SPI9-14, SPI16-17, SPI19, SPI23, SPI24	positive	11
H122800341	nited Kingdor	2012	Human	SRR1635115	NA	SPI1-6, SPI9-14, SPI16-17, SPI19, SPI23, SPI24	positive	11
H122820788	nited Kingdor	2012	Human	SRR1645641	NA	SPI1-6, SPI9-14, SPI16-17, SPI19, SPI23, SPI24	positive	11
H122880621	nited Kingdor	2012	Human	SRR1646224	NA	SPI1-6, SPI9-14, SPI16-17, SPI19, SPI23, SPI24	positive	11
H123280504	nited Kingdor	2012	Human	SRR1645738	NA	SPI1-6, SPI9-14, SPI16-17, SPI19, SPI23	positive	11
H123540624	nited Kingdor	2012	Human	SRR1646123	NA	SPI1-6, SPI9-14, SPI16-17, SPI19, SPI23, SPI24	positive	11
H123740526	nited Kingdor	2012	Human	SRR1646393	NA	SPI1-6, SPI9-14, SPI16-17, SPI19, SPI23, SPI24	positive	11
H124180493	nited Kingdor	2012	Human	SRR1646223	NA	SPI1-6, SPI9-14, SPI16-17, SPI19, SPI23, SPI24	positive	11
H124220645	nited Kingdor	2012	Human	SRR5814208	NA	SPI1-6, SPI9-14, SPI16-17, SPI19, SPI23	positive	183
H124420608	nited Kingdor	2012	Human	SRR1646252	NA	SPI1-6, SPI9-14, SPI16-17, SPI19, SPI23, SPI24	positive	11
H130220269	nited Kingdor	2013	Human	SRR1646083	NA	SPI1-6, SPI9-14, SPI16-17, SPI19, SPI23, SPI24	positive	11
CFSAN029318	United States	2015	Stool	SRR1805601	NA	SPI1-6, SPI9-14, SPI16-17, SPI19, SPI23, SPI24	positive	11
CFSAN029532	United States	2015	Stool	SRR1828977	NA	SPI1-6, SPI9-14, SPI16-17, SPI19, SPI23, SPI24	positive	11
CFSAN030338	United States	2015	Stool	SRR1982188	NA	SPI1-6, SPI9-14, SPI16-17, SPI19, SPI23, SPI24	positive	11

CFSAN030342	United States	2015	Stool	SRR1982124	NA	SPI1-6, SPI9-14, SPI16-17, SPI19, SPI23, SPI24	positive	11
CFSAN032432	United States	2015	Stool	SRR1998961	NA	SPI1-6, SPI9-14, SPI16-17, SPI19, SPI23, SPI24	positive	11
CFSAN032798	United States	2015	Stool	SRR2035441	NA	SPI1-6, SPI9-14, SPI16-17, SPI19, SPI23, SPI24	positive	11
CFSAN033186	United States	2015	Urine	SRR2035447	NA	SPI1-6, SPI9-14, SPI16-17, SPI19, SPI23, SPI24	positive	11
CFSAN033727	United States	2015	Stool	SRR2047358	NA	SPI1-6, SPI9-14, SPI16-17, SPI19, SPI23, SPI24	positive	11
CFSAN033954	United States	2015	Stool	SRR2047362	NA	SPI1-6, SPI9-14, SPI16-17, SPI19, SPI23, SPI24	positive	11
CFSAN033958	United States	2015	Stool	SRR2054257	NA	SPI1-6, SPI9-14, SPI16-17, SPI19, SPI23, SPI24	positive	11
CFSAN034156	United States	2015	Ascitic Fluid	SRR2056002	NA	SPI1-6, SPI9-14, SPI16-17, SPI19, SPI23, SPI24	positive	11
CFSAN034663	United States	2015	Stool	SRR2072484	NA	SPI1-6, SPI9-14, SPI16-17, SPI19, SPI23, SPI24	positive	11
CFSAN035607	United States	2015	Stool	SRR2102442	NA	SPI1-6, SPI9-14, SPI16-17, SPI19, SPI23, SPI24	positive	11
CFSAN046971	United States	2015	Blood	SRR3198650	NA	SPI1-6, SPI9-14, SPI16-17, SPI19, SPI23, SPI24	positive	11
CFSAN046972	United States	2015	Stool	SRR3198651	NA	SPI1-6, SPI9-14, SPI16-17, SPI19, SPI23, SPI24	positive	11
CFSAN046973	United States	2016	Stool	SRR3198652	NA	SPI1-6, SPI9-14, SPI16-17, SPI19, SPI23, SPI24	positive	11
CFSAN047797	United States	2015	Blood	SRR3219249	NA	SPI1-6, SPI9-14, SPI16-17, SPI19, SPI23, SPI24	positive	11
CFSAN047798	United States	2015	Stool	SRR3219250	NA	SPI1-6, SPI9-14, SPI16-17, SPI19, SPI23, SPI24	positive	11
CFSAN047799	United States	2015	Stool	SRR3224203	NA	SPI1-6, SPI9-14, SPI16-17, SPI19, SPI23, SPI24	positive	11
CFSAN047800	United States	2015	Stool	SRR3219120	NA	SPI1-6, SPI9-14, SPI16-17, SPI19, SPI23, SPI24	positive	11
CFSAN047801	United States	2015	Stool	SRR3219121	NA	SPI1-6, SPI9-14, SPI16-17, SPI19, SPI23, SPI24	positive	11
CFSAN047802	United States	2015	Stool	SRR3219123	NA	SPI1-6, SPI9-14, SPI16-17, SPI19, SPI23, SPI24	positive	11
CFSAN047803	United States	2015	Stool	SRR3219124	NA	SPI1-6, SPI9-14, SPI16-17, SPI19, SPI23, SPI24	positive	11
CFSAN047804	United States	2015	Stool	SRR3219125	NA	SPI1-6, SPI9-14, SPI16-17, SPI19, SPI23, SPI24	positive	11
CFSAN047805	United States	2015	Stool	SRR3219127	NA	SPI1-6, SPI9-14, SPI16-17, SPI19, SPI23, SPI24	positive	11
CFSAN047806	United States	2015	Stool	SRR3219133	NA	SPI1-6, SPI9-14, SPI16-17, SPI19, SPI23, SPI24	positive	11
CFSAN047807	United States	2015	Stool	SRR3219135	NA	SPI1-6, SPI9-14, SPI16-17, SPI19, SPI23, SPI24	positive	11
CFSAN038683	United States	2015	Stool	SRR2533493	NA	SPI1-6, SPI9-14, SPI16-17, SPI19, SPI23, SPI24	positive	11
CFSAN039269	United States	2015	Blood	SRR2559367	NA	SPI1-6, SPI9-14, SPI16-17, SPI19, SPI23, SPI24	positive	11
CFSAN040200	United States	2015	Blood	SRR2831441	NA	SPI1-6, SPI9-14, SPI16-17, SPI19, SPI23, SPI24	positive	11
CFSAN040203	United States	2015	Stool	SRR2831444	NA	SPI1-6, SPI9-14, SPI16-17, SPI19, SPI23, SPI24	positive	11

CFSAN047895	United States	2016	Stool	SRR3271876	NA	SPI1-6, SPI9-14, SPI16-17, SPI19, SPI23, SPI24	positive	11
CFSAN048622	United States	2016	Stool	SRR3372228	NA	SPI1-6, SPI9-14, SPI16-17, SPI19, SPI23, SPI24	positive	11
CFSAN051776	United States	2016	Stool	SRR3707420	NA	SPI1-6, SPI9-14, SPI16-17, SPI19, SPI23, SPI24	positive	11
CFSAN053061	United States	2016	Stool	SRR3990988	NA	SPI1-6, SPI9-14, SPI16-17, SPI19, SPI23, SPI24	positive	11
CFSAN053426	United States	2016	Stool	SRR4022300	NA	SPI1-6, SPI9-14, SPI16-17, SPI19, SPI23, SPI24	negative	11
MDH_2014_00245	United States	2012	Stool	SRR1188480	NA	SPI1-6, SPI9-14, SPI16-17, SPI19, SPI23, SPI24	negative	11
MDH_2014_00247	United States	2012	Arm Swab	SRR1188464	NA	SPI1-6, SPI9-14, SPI16-17, SPI19, SPI24	positive	11
MDH_2014_00248	United States	2013	Stool	SRR1188538	NA	SPI1-6, SPI9-14, SPI16-17, SPI19, SPI23, SPI24	positive	11
MDH_2014_00255	United States	2014	Stool	SRR1188508	NA	SPI1-6, SPI9-14, SPI16-17, SPI19, SPI23, SPI24	negative	11
MDH_2014_00256	United States	2014	Stool	SRR1188434	NA	SPI1-6, SPI9-14, SPI16-17, SPI19, SPI23, SPI24	negative	11
MDH_2014_00782	United States	2014	Stool	SRR1257318	NA	SPI1-6, SPI9-14, SPI16-17, SPI19, SPI23, SPI24	positive	11
MDH_2014_00789	United States	2014	Stool	SRR1263418	NA	SPI1-6, SPI9-14, SPI16-17, SPI19, SPI23, SPI24	positive	11
MDH_2014_00806	United States	2014	Urine	SRR1300694	NA	SPI1-6, SPI9-14, SPI16-17, SPI19, SPI23, SPI24	positive	11
MDH_2014_00808	United States	2014	Stool	SRR1302837	NA	SPI1-6, SPI9-14, SPI16-17, SPI19, SPI23, SPI24	positive	11
MDH_2014_00810	United States	2014	Stool	SRR1302865	NA	SPI1-6, SPI9-14, SPI16-17, SPI19, SPI23, SPI24	positive	11
CFSAN018241	United States	2014	Stool	SRR1334489	NA	SPI1-6, SPI9-14, SPI16-17, SPI19, SPI23, SPI24	positive	11
CFSAN022980	United States	2014	Urine	SRR1536148	NA	SPI1-6, SPI9-14, SPI16-17, SPI19, SPI23, SPI24	positive	11
CFSAN022981	United States	2014	Stool	SRR1536142	NA	SPI1-6, SPI9-14, SPI16-17, SPI19, SPI23, SPI24	positive	11
CFSAN024112	United States	2014	Stool	SRR1574286	NA	SPI1-6, SPI9-14, SPI16-17, SPI19, SPI23, SPI24	negative	11
NY_55235726	United States	2015	Stool	SRR1979446	NA	SPI1-6, SPI9-14, SPI16-17, SPI19, SPI23, SPI24	positive	11
NY_swgs1356	United States	2014	Wound	SRR1521521	NA	SPI1-6, SPI9-14, SPI16-17, SPI19, SPI23, SPI24	positive	11
CFSAN025251	United States	2014	Stool	SRR1633506	NA	SPI1-6, SPI9-14, SPI16-17, SPI19, SPI23, SPI24	positive	11
NY56657591	United States	2015	Stool	SRR2085497	NA	SPI1-6, SPI9-14, SPI16-17, SPI19, SPI23, SPI24	positive	11
NY58277053	United States	2015	Stool	SRR2218462	NA	SPI1-6, SPI9-14, SPI16-17, SPI19, SPI23, SPI24	positive	11
NY58474062	United States	2015	Stool	SRR2296842	NA	SPI1-6, SPI9-14, SPI16-17, SPI19, SPI23, SPI24	positive	11
NY59080619	United States	2015	Stool	SRR2847920	NA	SPI1-6, SPI9-14, SPI16-17, SPI19, SPI23, SPI24	positive	11
NY59297727	United States	2015	Body Fluid	SRR2891000	NA	SPI1-6, SPI9-14, SPI16-17, SPI19, SPI23, SPI24	positive	11
NY63966472	United States	2016	Stool	SRR3457611	NA	SPI1-6, SPI9-14, SPI16-17, SPI19, SPI23, SPI24	positive	11

CFSAN047178	United States	2016	Stool	SRR3215231	NA	SPI1-6, SPI9-14, SPI16-17, SPI19, SPI23, SPI24	positive	11
CFSAN050983	United States	2016	Stool	SRR3606935	NA	SPI1-6, SPI9-14, SPI16-17, SPI19, SPI23, SPI24	positive	11
CFSAN052649	United States	2016	Stool	SRR3824012	NA	SPI1-6, SPI9-14, SPI16-17, SPI19, SPI23, SPI24	positive	11
CFSAN053020	United States	2016	Stool	SRR3944461	NA	SPI1-6, SPI9-14, SPI16-17, SPI19, SPI23, SPI24	positive	11
CFSAN055194	United States	2016	Stool	SRR4256079	NA	SPI1-6, SPI9-14, SPI16-17, SPI19, SPI23, SPI24	positive	11
CFSAN056386	United States	2016	Stool	SRR5058013	NA	SPI1-6, SPI9-14, SPI16-17, SPI19, SPI23, SPI24	positive	11
CFSAN056389	United States	2016	Urine	SRR5057975	NA	SPI1-6, SPI9-14, SPI16-17, SPI19, SPI23, SPI24	positive	11
CFSAN056391	United States	2016	Stool	SRR5057976	NA	SPI1-6, SPI9-14, SPI16-17, SPI19, SPI23, SPI24	positive	11
CFSAN058045	United States	2014	Stool	SRR5139134	NA	SPI1-6, SPI9-14, SPI16-17, SPI19, SPI23, SPI24	positive	11
CFSAN058048	United States	2014	Stool	SRR5139133	NA	SPI1-6, SPI9-14, SPI16-17, SPI19, SPI23, SPI24	positive	11
CFSAN058050	United States	2014	Stool	SRR5139183	NA	SPI1-6, SPI9-14, SPI16-17, SPI19, SPI23, SPI24	positive	11
CFSAN058057	United States	2014	Stool	SRR5139184	NA	SPI1-6, SPI9-14, SPI16-17, SPI19, SPI23, SPI24	positive	11
CFSAN058115	United States	2014	Stool	SRR5152277	NA	SPI1-6, SPI9-14, SPI16-17, SPI19, SPI23, SPI24	positive	11
CFSAN058116	United States	2014	Stool	SRR5152274	NA	SPI1-6, SPI9-14, SPI16-17, SPI19, SPI23, SPI24	positive	11
CFSAN059116	United States	2016	Stool	SRR5182245	NA	SPI1-6, SPI9-14, SPI16-17, SPI19, SPI23, SPI24	positive	11
CFSAN059117	United States	2016	Stool	SRR5182250	NA	SPI1-6, SPI9-14, SPI16-17, SPI19, SPI23, SPI24	positive	11
swgs1025	United States	2013	Stool	SRR1187594	NA	SPI1-6, SPI9-14, SPI16-17, SPI19, SPI23, SPI24	positive	11
swgs1062	United States	2013	Blood	SRR1187422	NA	SPI1-6, SPI9-14, SPI16-17, SPI19, SPI23, SPI24	positive	11
swgs1069	United States	2013	Stool	SRR1187438	NA	SPI1-6, SPI9-14, SPI16-17, SPI19, SPI23, SPI24	negative	11
swgs1079	United States	2013	Stool	SRR1187417	NA	SPI1-6, SPI9-14, SPI16-17, SPI19, SPI23, SPI24	positive	11
swgs1194	United States	2013	Stool	SRR1187596	NA	SPI1-6, SPI9-14, SPI16-17, SPI19, SPI23, SPI24	positive	11
CFSAN072891	United States	2013	Stool	SRR6377133	NA	SPI1-6, SPI9-14, SPI16-17, SPI19, SPI23	positive	11
CFSAN072892	United States	2013	Stool	SRR6377123	NA	SPI1-6, SPI9-14, SPI16-17, SPI19, SPI23	positive	11
CFSAN072894	United States	2013	Stool	SRR6377127	NA	SPI1-6, SPI9-14, SPI16-17, SPI19, SPI23	positive	11
CFSAN072902	United States	2013	Stool	SRR6377031	NA	SPI1-6, SPI9-14, SPI16-17, SPI19, SPI23	positive	*
CFSAN072909	United States	2013	Stool	SRR6411492	NA	SPI1-6, SPI9-14, SPI16-17, SPI19, SPI23	positive	11
CFSAN072936	United States	2014	Stool	SRR7749331	NA	SPI1-6, SPI9-14, SPI16-17, SPI19, SPI23	positive	11
CFSAN072938	United States	2014	Stool	SRR6491046	NA	SPI1-6, SPI9-14, SPI16-17, SPI19, SPI23	positive	11

CFSAN072939	United States	2014	Stool	SRR6498587	NA	SPI1-6, SPI9-14, SPI16-17, SPI19, SPI23	positive	11
WAPHL_SAL_A00615	United States	2014	Stool	SRR1576209	NA	SPI1-6, SPI9-14, SPI16-17, SPI19, SPI23, SPI24	positive	11
CFSAN023725	United States	2014	Stool	SRR1576023	NA	SPI1-6, SPI9-14, SPI16-17, SPI19, SPI23, SPI24	positive	11
CFSAN024924	United States	2014	Stool	SRR1615109	NA	SPI1-6, SPI9-14, SPI16-17, SPI19, SPI23, SPI24	positive	11
CFSAN024929	United States	2014	Stool	SRR1615087	NA	SPI1-6, SPI9-14, SPI16-17, SPI19, SPI23, SPI24	positive	11
CFSAN024932	United States	2014	Stool	SRR1615104	NA	SPI1-6, SPI9-14, SPI16-17, SPI19, SPI23, SPI24	positive	11
CFSAN027409	United States	2014	Stool	SRR1709664	NA	SPI1-6, SPI9-14, SPI16-17, SPI19, SPI23, SPI24	positive	11
CFSAN030353	United States	2014	Stool	SRR1973913	NA	SPI1-6, SPI9-14, SPI16-17, SPI19, SPI23, SPI24	positive	11
CFSAN030354	United States	2014	Stool	SRR1973686	NA	SPI1-6, SPI9-14, SPI16-17, SPI19, SPI23, SPI24	positive	11
CFSAN030355	United States	2015	Stool	SRR1973786	NA	SPI1-6, SPI9-14, SPI16-17, SPI19, SPI23, SPI24	positive	11
CFSAN039202	United States	2015	Stool	SRR2648211	NA	SPI1-6, SPI9-14, SPI16-17, SPI19, SPI23, SPI24	positive	11
MLI21	Mali	2002	Blood	ERR025166	NA	SPI1-6, SPI9-14, SPI16-17, SPI19, SPI24	positive	11
MLP43	Mali	2005	Blood	ERR025174	NA	SPI1-6, SPI9-14, SPI16-17, SPI19, SPI24	positive	11
31_88	Uruguay	1988	Gastroenteritis	ERR036132	NA	SPI1-6, SPI9-14, SPI16-17, SPI19, SPI23, SPI24	positive	1974
08_89	Uruguay	1989	Blood	ERR036134	NA	SPI1-6, SPI9-14, SPI16-17, SPI19, SPI23, SPI24	positive	1974
8_02	Uruguay	2002	Stool	ERR036138	NA	SPI1-6, SPI9-14, SPI16-17, SPI19, SPI23, SPI24	positive	11
214_02	Uruguay	2002	Blood	ERR036139	NA	SPI1-6, SPI9-14, SPI16-17, SPI19, SPI23, SPI24	negative	11
10_05	Uruguay	2005	Stool	ERR036140	NA	SPI1-6, SPI9-14, SPI16-17, SPI19, SPI23, SPI24	positive	11
KUMAR19	nited Kingdor	2009	Stool	ERR048520	NA	SPI1-6, SPI9-14, SPI16-17, SPI19, SPI23, SPI24	positive	11
A59040	Malawi	2010	Blood	ERR235154	NA	SPI1-6, SPI9-14, SPI16-17, SPI19, SPI24	positive	11
A51073	Malawi	2008	Blood	ERR235181	NA	SPI1-6, SPI9-14, SPI16-17, SPI19, SPI23, SPI24	positive	11
32863	Congo	2011	Blood	ERR235296	NA	SPI1-6, SPI9-14, SPI16-17, SPI19, SPI24	positive	11
33013	Congo	2011	Blood	ERR235298	NA	SPI1-6, SPI9-14, SPI16-17, SPI19, SPI24	positive	11
34233	Congo	2011	Blood	ERR235323	NA	SPI1-6, SPI9-14, SPI16-17, SPI19, SPI24	positive	11
1020983	Malawi	2011	Blood	ERR235380	NA	SPI1-6, SPI9-14, SPI16-17, SPI19, SPI24	positive	11
SAEN4582	Uganda	1997	Blood	ERR311309	NA	SPI1-6, SPI9-14, SPI16-17, SPI19, SPI23, SPI24	positive	11
463134	South Africa	2010	Blood	ERR338905	NA	SPI1-6, SPI9-14, SPI16-17, SPI19, SPI23, SPI24	positive	11
177883	South Africa	2007	Blood	ERR338920	NA	SPI1-6, SPI9-14, SPI16-17, SPI19, SPI23, SPI24	positive	11

516364	South Africa	2011	Blood	ERR338923	NA	SPI1-6, SPI9-14, SPI16-17, SPI19, SPI23, SPI24	positive	11
06_6063	Senegal	2006	Blood	ERR369359	NA	SPI1-6, SPI9-14, SPI16-17, SPI19, SPI23, SPI24	positive	11
201007599	Mali	2010	Blood	ERR369418	NA	SPI1-6, SPI9-14, SPI16-17, SPI19, SPI24	positive	11
W47	Mali	2012	NA	ERR374229	NA	SPI1-6, SPI9-14, SPI16-17, SPI19, SPI24	positive	11
V91	Mali	2011	NA	ERR374236	NA	SPI1-6, SPI9-14, SPI16-17, SPI19, SPI24	positive	11

Table S2. Clade II specific genes and assigned gene-ontology terms.

Gene names in Prokka	Clade II specific	Product	Biological Process	Molecular Function
adaA	all	Bifunctional transcriptional activator/DNA repair enzyme AdaA	GO:0033554, GO:0006259	GO:0016740, GO:0003676, GO:0043167
ampC	S211	blaCMY-2	GO:0017001, GO:0046677	GO:0016787
appA	all	Oligopeptide-binding protein AppA	GO:0055085, GO:0015833	GO:0005215
arfA	all	Alternative ribosome-rescue factor A	GO:0006414	GO:0003676
ariR	not in 201600169	putative two-component-system connector protein AriR	GO:0071468, GO:0044010, GO0042542	GO:0003676
bdbD	all	Disulfide bond formation protein D	GO:0030420	GO:0016491
bepE	S104, S109, S74, S95	Efflux pump membrane transporter BepE	GO:0055085	GO:0015562
besA	all	Ferri-bacillibactin esterase BesA	N/A	GO:0052689
betC	all	Choline-sulfatase	GO:0042425	GO:0016787, GO:0043167
bfpA	not in 201600169, 201607119, 201607559	Major structural subunit of bundle-forming pilus	N/A	N/A
bpoA1	all	Non-heme chloroperoxidase CPO-A1	GO:0098869, GO:0017000	GO:0004601
casA	all	CRISPR system Cascade subunit CasA	GO:0051607	GO:003676, GO:0046872
casD	all	CRISPR system Cascade subunit CasD	GO:0051607, GO:0006259	GO:003676
cat1	all	Succinyl-CoA:coenzyme A transferase	GO:006082, GO:006084	GO:0016740
cbh	all	Choloylglycine hydrolase	GO:0006699, GO:0006629	GO:0016787
ccmK1	all	Carbon dioxide-concentrating mechanism protein CcmK	GO:0015977	N/A
ccmK2	all	Carbon dioxide-concentrating mechanism protein CcmK	GO:0015977	N/A
ccp	all	Cytochrome c551 peroxidase	GO:0098869	GO:0004601
chiA	all	Chitinase A	GO:0006032, GO:0000272	GO:0016787, GO:0008061
cia	S211	Colicin-Ia	GO:0042742, GO:0019835	N/A
clcD	all	Carboxymethylenebutenolidase	GO:0019439	GO:0016787
comEA	all	ComE operon protein 1	GO:0015628, GO:0030420, GO:0006281	GO:0003676
comEC	all	ComE operon protein 3	GO:0030420	N/A
cpo	all	Non-heme chloroperoxidase	GO:0098869, GO:0017000	GO:0004601
dhfrI	S216	Dihydrofolate reductase type 1	GO:0046654, GO:0006730, GO:0042221	GO:0016491, GO:0000166
dpnA	S74	Modification methylase DpnIIB	GO:0006306	GO:0016740, GO:0003676
dprA	all	DNA processing protein DprA	GO:0039420	GO:0003676
eae	all	Intimin	GO:0007155	N/A
ecoRIIR	CFSAN030342, NY_swgs1356	Type-2 restriction enzyme EcoRII	GO:0090304	GO:0016787, GO:0004518, GO:0003676
entD	all	Enterobactin synthase component D	GO:0006464, GO:0009239	GO:0046872, GO:0008897, GO:0016740

epsL	not in 201600169, 201607119	putative sugar transferase EpsL	N/A	GO:0016740
exsA	all	Exoenzyme S synthesis regulatory protein ExsA	GO:0006355	GO:0140110, GO:0003676
fimA	all	Fimbrial subunit type 1	GO:0007155	GO:0005198
fliV	all	Flagellar biosynthetic protein FliV	GO:0044781	N/A
folA	all	Dihydrofolate reductase	GO:0042221, GO:0009410	GO:0016491, GO:0000166, GO:0043167, GO:0005542
fosA	S104	Glutathione transferase FosA	GO:0042221	GO:0016740, GO:0043167
gadW	all	HTH-type transcriptional regulator GadW	GO:0006355	GO:0140110, GO:0003676
gspA	not in 201600169, 201607119, 201607559	General stress protein A	N/A	GO:0016740, GO:0043167, GO:0000166
hcpD	all	Putative beta-lactamase HcpD	GO:0042221	GO:0016787
hsdR	in 31 isolates	Type I restriction enzyme EcoR124II R protein	GO:0090304	GO:0003676, GO:0016787, GO:0004518, GO:0004386,
hxlR	all	HTH-type transcriptional activator HxlR	N/A	GO:0003676
icaB	all	Poly-beta-1,6-N-acetyl-D-glucosamine N-deacetylase	GO:0005975	GO:0016787
kanE	all	Alpha-D-kanosaminyltransferase	GO: 1901133	GO:0016740
kcsA	all	pH-gated potassium channel KcsA	GO:0071805, GO:0005249	GO:0005515
lppC	all	Putative lipoprotein LppC	N/A	N/A
ltrA	H122820788	Group II intron-encoded protein LtrA	GO:0071897, GO:0090304	GO:0016772, GO:0016787
malX	all	PTS system maltose-specific EIICB component	GO:0098704, GO:0034219	GO:0005215, GO:0016772, GO:0005515
mcpQ	all	Methyl-accepting chemotaxis protein McpQ	GO:0006935, GO:0007165	N/A
meh	not in 201600169, 201607119	Mesaconyl-C(4)-CoA hydratase	N/A	N/A
merA	S216	Mercuric reductase	GO:0050787, GO:0045454	GO:0016491, GO:0043167, GO:0000166
merC	S216	Mercuric transport protein MerC	GO:0015694, GO:0046689	GO:0043167
merP	S216	Mercuric transport protein periplasmic component	GO:0015694, GO:0046689	GO:0043167, GO:0015075
merT	S216	Mercuric transport protein MerT	GO:0015694, GO:0046689	GO:0043167, GO:0015075
mftF	not in 201600169, 201607119, 201607559, S214	Putative mycofactocin biosynthesis glycosyltransferase MftF	N/A	GO:0016740
moeZ	all	putative adenylyltransferase/sulfurtransferase	GO:0019344	GO:0016740, GO:0003824, GO:0000166
neo	S104, 109, S181, S189, S204, S225, S74, S95	Aminoglycoside 3'-phosphotransferase	GO:0016310, GO:0046677	GO:0016740, GO:0000166
oatA	not in 201600169, 201607119, 201607559, S216	O-acetyltransferase OatA	GO:0009103	GO:0016740
pasI	all	Persistence and stress-resistance antitoxin PasI	pasT/pasI toxin-antitoxin system	
pchR	all	Regulatory protein PchR	GO:0006355	GO:0003676
pemI	06_6063, S104, S109, S181, S189, S204, S225, S74, S95	Antitoxin PemI	N/A	GO:0003676
pemK	06_6063, S104, S109, S181, S189, S204, S225, S74, S95	Endoribonuclease PemK	GO:0090305	GO:0003676, GO:0016787
pgaB	all	Poly-beta-1, 6-N-acetyl-D-glucosamine N-deacetylase	GO:0098732	GO:0016787

pifC	06_6063	Transcriptional repressor PifC	N/A	N/A
pilE1	all	Fimbrial protein	GO:0007155	N/A
pndA	06_6063, H122820788, S211, S81	Protein PndA	toxic component in TA system	
prn	not in 190768, 201600169, 201607119, 201607559, S214,	Pertactin autotransporter	GO:0007155	GO:0005515
prtR	H120980502	HTH-type transcriptional regulator PrtR	GO:0030152, GO:1900377	GO:0003676
pstS1	all	Phosphate-binding protein PstS 1	GO:0055085, GO:0009267, GO:0006811	GO:0005515, GO:0043167
repE	S74, NY_sgws1356, CFSAN030342	Replication initiation protein	GO:0006260, GO:0071897	GO:0034061, GO:0003676
rffH	all	Glucose-1-phosphate thymidylyltransferase 2	GO:0045226	GO:0016740, GO:0043167, GO:0005515
rsr	all	60 kDa SS-A/Ro ribonucleoprotein	N/A	GO:0005515, GO:0003676, GO:0043167
rutB	H122060292	Peroxyureidoacrylate/ureidoacrylate amidohydrolase RutB	GO:0019740	GO:0016787
sadH	all	Putative oxidoreductase SadH	GO:0009268	GO:0016491
sfp	all	4'-phosphopantetheinyl transferase sfp	GO:0009085, GO:0017000, GO:0036211	GO:0016740, GO:0043167
siaQ	all	Sialic acid TRAP transporter small permease protein SiaQ	GO:0008643	GO:0022857
spo0C	13 isolates	Chromosome-partitioning protein Spo0J	N/A	N/A
tlyC	all	Hemolysin C	GO:0019835	GO:0050660, GO:0090729
tnpA	S104, 109, S181, S189, S204, S225, S74, S95	Transposase for transposon Tn5	GO:0006355	GO:0003676
torI	H120980502	Response regulator inhibitor for tor operon	GO:2000143, GO:0006259	GO:0005515, GO:0003676
tufA	in 21 isolates	Elongation factor Tu 1	GO:0006414	GO:0016787, GO:0005515, GO:0000166, GO:0003746
virB1	50129, S216	Type IV secretion system protein VirB11	GO:0032940	GO:0005524, GO:0000166
wfgD	all	UDP-Glc:alpha-D-GlcNAc-diphosphoundecaprenol beta-1,3-glucosyltransferase WfgD	GO:0009103	GO:0016740
yafN	CFSAN030338	Antitoxin YafN	GO:0009432, GO:0044092, GO:0006355	GO:0003676, GO:0005515
ycdT	all	putative diguanylate cyclase YcdT	GO:0042710,	GO:0016740, GO:0000166, GO:0005525, GO:0043167
yefM	all	Antitoxin YefM	GO:0045892, GO:0042710	GO:0003676, GO:0015643
yegE	all	putative diguanylate cyclase YegE	N/A	GO:0016740, GO:0005525, GO:0000166, GO:0043167
ygaZ	all	Inner membrane protein YgaZ	GO:1903785	N/A
yhdN	all	General stress protein 69	GO:0007165	GO:0000155, GO:0016491, GO:0005515
yhjX	all	putative MFS-type transporter YhjX	GO:0055085, GO:0031669	GO:0022857
yiaB	all	Inner membrane protein YiaB	GO:0033554	N/A
yokD	S95	SPBc2 prophage-derived aminoglycoside N(3')-	GO:0046677	GO:0016740
yvqK	all	Cob(I)yrinic acid a, c-diamide adenosyltransferase	GO:00009236	GO:0016740, GO:0000166, GO:0005524
ywaD	all	Aminopeptidase YwaD	GO:0019538	GO:0008233

Table S3. Prevalence of prophages identified by PHASTER among these S. Enteritidis human isolates.

Isolates	Acinet_AM101_NC_049511	Acinet_vB_ApiM_fHyAci03_NC_049438	Acinet_YMC11/11/R3177_NC_041866	Burkho_BcepMu_NC_005882	Cronob_ENT39118_NC_019934	Cronob_ESSI_2_NC_047854	Edward_GF_2_NC_026611	Entero_lambda_NC_001416	Entero_mEp237_NC_019704	Entero_P1_NC_005856	Entero_phiP27_NC_003356	Entero_phiT5282H_NC_049429	Erwini_PEp14_NC_016767	Erwini_vB_EamM_ChrisDB_NC_031126	Erwini_vB_EamP_S6_NC_019514	Escher_500465_1_NC_049342	Escher_500465_2_NC_049343	Escher_phi191_NC_028660	Escher_RCS47_NC_042128	Escher_SH2026Stx1_NC_049919	Klebsi_Seifer_NC_048732	Gifsy_1_NC_010392	Gifsy_2_NC_010393	Rhodoc_Takoda_NC_048708	Rhodof_P26218_NC_029061	Salmon_118970_sal3_NC_031940	Fels_1_NC_010391	Fels_2_NC_010463	Salmon_g341c_NC_013059	RE_2010_NC_019488	SEN34_NC_028699	SEN8_NC_047753	SJ46_NC_031129	vB_SosS_Oslo_NC_018279	Shigel_POCJ13_NC_025434	Shigel_SfIV_NC_022749	Shigel_Stx_NC_029120	SETP7_NC_022754	Total
CFSAN029318																	+						+							+			+			+	+		6
CFSAN029532																	+						+			+							+		+	+			6
CFSAN030338																	+						+			+							+		+	+			6
CFSAN030342																	+						+			+							+				+		5
CFSAN032432																	+						+			+							+			+	+		6
CFSAN032798																							+			+							+			+	+		5
CFSAN033186																	+						+			+							+			+	+		6
CFSAN033727						+											+						+							+			+		+	+			7
CFSAN033954						+											+						+							+			+		+				6
CFSAN033958																							+			+							+		+	+			5
CFSAN034156																	+						+							+			+				+		5
CFSAN034663						+											+						+				+			+			+		+				7
CFSAN035607																	+						+							+			+		+	+			6
16EP000866																	+						+							+			+		+				5
16EP001008																	+						+							+			+		+				5
16EP001290																	+						+							+			+		+				5
16EP001384																	+						+							+			+		+				5
16EP001503																	+						+							+			+			+	+		6
16EP002245																	+						+							+			+				+		5
5795																	+						+				+			+			+			+	+		7
5946																	+						+							+			+			+	+		6
6012																	+						+							+			+		+	+			6
6175																	+						+							+			+			+	+		6
6388																	+		+				+							+					+	+			6
37009							+										+					+	+			+		+	+							+			8
37045																	+						+							+			+			+	+		6
45605																	+						+							+			+		+	+			6
49692																	+						+							+			+			+			5
49740						+											+					+	+										+			+	+		7
50129																							+																1
60143																	+									+							+		+	+			5
62513																	+						+			+							+		+	+			6
133218							+										+					+	+			+						+				+			7
137161																	+						+							+			+			+	+		6
138357																	+						+			+						+	+			+	+		7
161872																	+						+							+			+			+	+		6
173732							+										+					+	+			+	+	+								+			8
182120																	+						+							+			+			+	+		6
182121																	+						+							+			+			+	+		6
190566																	+						+							+			+			+	+		6
190768																	+						+			+							+		+	+			6
273546																	+						+			+						+	+		+	+			7
273568																	+					+	+			+	+	+								+			7
278106																	+						+							+			+		+	+			6
278154																	+						+							+					+	+			5
289642																						+	+				+						+		+	+			6
293745																	+					+	+										+		+	+			6
313428																	+						+			+							+		+	+			6
323071																	+						+			+							+		+	+			6
497131																							+							+			+			+	+		5
500262																	+						+				+			+			+			+			6
503131															+		+						+										+			+			5
201405122																	+						+							+			+			+	+		6
201405756																	+						+							+			+			+	+		6
201405757																	+						+							+			+		+				5
201405760																	+						+							+			+				+		5
201405861																	+						+							+			+			+	+		6
201600169																										+													1
201607119																										+							+						2
201607559																										+							+						2
201608919																	+						+				+						+						4
CFSAN046971																	+						+							+					+	+			5
CFSAN046972																	+						+							+			+			+	+		6
CFSAN046973																	+						+							+			+		+	+			6
CFSAN047797																	+						+							+			+				+		5
CFSAN047798																	+						+							+			+		+	+			6
CFSAN047799																	+						+							+			+		+				5
CFSAN047800																							+							+			+		+				4
CFSAN047801																	+						+							+			+			+	+		6
CFSAN047802																	+						+							+			+				+		5
CFSAN047803																	+						+							+			+		+				5
CFSAN047804																	+						+							+			+				+		5
CFSAN047805																	+						+							+			+		+				5
CFSAN047806																	+						+							+			+		+				5
CFSAN047807																	+						+							+			+		+	+			6
CFSAN038683																	+						+							+			+		+	+			6
CFSAN039269									+								+						+							+			+			+	+		7
CFSAN040200						+											+						+							+			+		+	+			7
CFSAN040203						+											+						+							+			+			+	+		7
CFSAN047895																	+						+							+			+			+	+		6
CFSAN048622																	+						+			+							+		+	+			6
CFSAN051776																	+					+	+							+			+			+	+		7

CFSAN053061																	+						+							+			+				+		5
CFSAN053426						+											+						+							+					+	+			6
H120980502																	+						+			+							+	+	+	+			7
H122060292																	+						+			+							+		+	+			6
H122280366																	+						+							+			+		+	+			6
H122800341																	+						+			+							+				+		5
H122820788													+				+			+			+			+							+		+	+			8
H122880621																	+						+			+						+	+		+	+			7
H123280504									+														+				+			+			+			+			6
H123540624																	+									+							+			+	+		5
H123740526								+									+						+			+							+		+	+			7
H124180493																	+						+			+							+			+	+		6
H124220645							+										+					+	+			+		+	+										7
H124420608																	+						+			+							+		+	+			6
H130220269																	+						+			+							+			+	+		6
MDH_2014_00245																	+						+							+						+	+		5
MDH_2014_00247											+	+					+					+	+				+						+			+			8
MDH_2014_00248																	+						+			+							+			+	+		6
MDH_2014_00255																	+						+							+						+	+		5
MDH_2014_00256																	+						+							+						+	+		5
MDH_2014_00782																	+						+			+							+			+	+		6
MDH_2014_00789																	+						+							+			+			+	+		6
MDH_2014_00806						+											+						+							+			+			+	+		7
MDH_2014_00808																	+						+							+			+			+	+		6
MDH_2014_00810						+											+						+							+			+				+		6
CFSAN018241																	+						+							+			+		+				5
CFSAN022980																	+						+							+			+				+		5
CFSAN022981																	+						+							+			+				+		5
CFSAN024112																	+						+							+					+	+			5
NY_S5235726																	+						+		+					+						+	+		6
NY_swgs1356																	+						+			+							+			+	+		6
CFSAN025251																	+						+			+							+		+	+			6
NY56657591																	+						+			+	+						+			+	+		7
NY58277053					+	+																	+							+						+	+		6
NY58474062																	+						+							+					+	+	+		6
NY59080619																							+				+			+			+		+				5
NY59297727																	+						+							+			+		+				5
NY63966472																	+						+							+			+		+	+			6
CFSAN047178						+																	+							+			+				+		5
CFSAN050983																							+							+	+				+				4
CFSAN052649																	+						+							+			+			+	+		6
CFSAN053020						+											+						+							+			+		+	+			7
CFSAN055194		+															+						+							+			+			+	+		7
CFSAN056386																	+						+				+			+			+				+		6
CFSAN056389																	+						+							+			+		+				5
CFSAN056391														+			+						+							+			+				+		6
CFSAN058045																							+							+			+		+	+			5
CFSAN058048																	+						+							+			+			+	+		6
CFSAN058050																	+						+							+			+			+	+		6
CFSAN058057																	+						+							+			+		+	+			6
CFSAN058115																	+						+							+			+		+	+			6
CFSAN058116			+														+						+							+			+			+			6
CFSAN059116											+						+						+				+			+			+		+				7
CFSAN059117																	+						+							+			+		+				5
S74																+	+				+		+			+													5
S81											+					+	+	+					+			+													6
S95											+						+						+			+													4
S104																	+				+		+			+													4
S109																	+				+		+			+													4
S181																	+						+			+							+		+	+			6
S183																	+						+			+	+						+			+	+		7
S189																	+						+			+							+			+	+		6
S190														+									+			+		+					+			+	+		7
S204	+																+						+			+	+						+				+		7
S205																	+						+			+										+	+		5
S209																+	+						+			+							+		+	+			7
S211										+							+						+	+		+							+			+	+		8
S214																	+						+			+							+				+		5
S216		+															+						+			+							+		+	+			7
S225																							+		+	+	+						+						5
swgs1025																	+						+							+			+		+	+			6
swgs1062																							+			+							+			+	+		5
swgs1069																	+						+							+					+	+			5
swgs1079						+									+		+						+							+			+			+	+		8
swgs1194																	+						+							+			+		+	+			6
CFSAN072891																	+						+							+			+			+	+		6
CFSAN072892																	+						+							+			+		+	+			6
CFSAN072894																	+						+							+			+			+	+		6
CFSAN072902																							+							+			+		+	+			5
CFSAN072909						+																	+							+			+			+	+		6
CFSAN072936						+																	+			+		+					+		+	+			7
CFSAN072938																							+			+							+			+	+		5
CFSAN072939																							+							+			+			+	+		5
WAPHL_SAL-A00615						+											+						+							+			+		+	+			7
CFSAN023725						+											+						+							+			+		+	+			7
CFSAN024924						+											+						+							+			+			+	+		7
CFSAN024929						+											+						+							+			+		+				6

CFSAN024932																	+						+							+			+			+	+		6
CFSAN027409																	+						+							+						+	+		5
CFSAN030353						+											+						+							+			+		+	+			7
CFSAN030354						+											+						+							+			+		+	+			7
CFSAN030355																	+						+							+			+		+	+			6
CFSAN039202						+																	+							+			+			+	+		6
MLI21												+					+					+											+			+			5
MLP43												+					+					+											+			+			5
201007599												+					+					+											+			+			5
W47												+					+					+											+			+			5
V91												+					+					+											+			+			5
8_02																	+						+			+							+			+			5
214_02																	+						+			+										+			4
10_05																	+						+			+							+			+			5
A51073																	+						+			+										+			4
177883																	+						+			+							+			+			5
06_6063																	+						+			+							+			+			5
31_88							+										+					+	+								+		+			+			7
08_89							+										+					+	+								+		+			+			7
KUMAR19																	+						+							+			+			+			5
SAEN4582																	+						+										+			+			4
463134						+											+						+							+			+			+			6
516364						+											+						+							+			+			+			6
A59040												+					+					+										+	+			+		+	6
32863												+					+					+										+				+			5
33013												+					+					+										+				+			5
34233												+					+					+										+				+			5
1020983												+					+					+	+									+				+			6
Total	1	2	1	0	1	24	6	1	2	1	4	11	1	2	2	3	173	1	1	1	3	22	183	1	2	63	16	6	2	114	3	9	164	1	75	148	79	1	
